# Copper(II)-Complexed Polyethylenimine-Entrapped Gold Nanoparticles Enable Targeted CT/MR Imaging and Chemodynamic Therapy of Tumors

**DOI:** 10.3390/polym17030423

**Published:** 2025-02-06

**Authors:** Lingxiu He, Na Liu, Risong Pan, Jingyi Zhu

**Affiliations:** 1 School of Pharmaceutical Sciences, Nanjing Tech University, Nanjing 211816, China; lingxiuhe@njtech.edu.cn (L.H.); 17756880765@163.com (N.L.); 2 College of Biotechnology and Pharmaceutical Engineering, Nanjing Tech University, Nanjing 211816, China; vty629@126.com

**Keywords:** Cu(II)-complexed polyethylenimine, gold nanoparticles, targeting, CT/MR imaging, chemodynamic therapy, cancer theranostics

## Abstract

Transition-metal ion copper(II) (Cu(II)) has drawn increasing attention as a small-molecular cancer theranostic agent. However, delivering a sufficient dosage of Cu(II) to the tumor site and integrating multiple imaging modalities to achieve precise and effective cancer theranostics remains a critical challenge. Herein, an emerging Cu(II)-based nanocomposite has been synthesized for targeted tumor computed tomography (CT)/magnetic resonance (MR) dual-mode imaging and chemodynamic therapy (CDT). Briefly, 2-picolinic acid (PA-COOH), polyethylene glycol (PEG)-linked folic acid (FA), and fluorescein isothiocyanate (FI) were sequentially conjugated with polyethylenimine (PEI.NH_2_) and then in situ fabrication of gold nanoparticles (Au NPs) occurred within the PEI.NH_2_ internal cavity. After acetylation of PEI.NH_2_ terminal amines and Cu(II) complexation, the Cu(II)-based nanocomposites FA-Au/Cu(II) PENPs with a mean diameter of 2.87 nm were generated. The synthesized FA-Au/Cu(II) PENPs showed favorable stability of colloidal dispersion, sustainable Cu(II) release properties in a pH-dependent manner, and Fenton-like catalytic activity specifically. With the FA-mediated targeting pathway, FA-Au/Cu(II) PENPs can specifically accumulate in cancer cells with high expression of FA receptors. Meanwhile, the complementary CT/MR dual-mode imaging in vitro and in vivo can be afforded by FA-Au/Cu(II) PENPs based on the excellent X-ray attenuation properties of Au NPs and the applicable r_1_ relaxivity (0.7378 mM^−1^s^−1^) of Cu(II). Notably, the Cu(II)-mediated CDT mechanism enables FA-Au/Cu(II) PENPs to elicit the generation of toxic hydroxyl radicals (·OH), depletion of glutathione (GSH), promotion of lipid peroxidation (LPO), and induction of cancer cell apoptosis in vitro, and further demonstrates remarkable anti-tumor efficacy in a xenograft tumor model. With the illustrated targeted theranostic capacity of FA-Au/Cu(II) PENPs towards tumors, this Cu(II)-based nanocomposite paradigm inspires the construction of advanced theranostic nanoplatforms incorporating alternative transition metal ions.

## 1. Introduction

The emergence and evolution of nanotechnology and nanoscience have facilitated advancements in oncological medicine, offering new perspectives for precise diagnosis and effective therapy of tumors [1,2,3,4]. In recent decades, numerous researchers have exhibited considerable interest in advanced theranostic nanomedicines, which simultaneously integrate diagnostic and therapeutic moieties in a unified nanosystem for multiple functionalities. These all-in-one theranostic nanoformulations hold promise for substantially enhancing treatment efficacy and potentially reducing the costs associated with tumor therapy [5,6,7,8]. Certainly, the innovative design, personalized customization, and performance optimization of theranostic nanoformulations have opened up a novel pathway for enhancing their bioavailability, tumor specificity, and theranostic efficacy, thus enabling real-time monitoring, comprehensive evaluation, and improved prognosis of tumors within the framework of advanced research [9,10,11,12,13]. However, the development and utilization of multifunctional theranostic nanoformulations are still constrained by several significant limitations, notably the intricate preparation procedures, lengthy production cycles, and complicated characterization methods in terms of nanoformulation synthesis [14,15]. Therefore, it is of great significance to employ simple modules with multifunctionality in the construction of theranostic nanoformulations to minimize the gap between diagnosis and therapy, thereby achieving synchronization.

Copper, a common trace element in the human body, has emerged as a hotspot in biomedical research owing to its tremendous potential for cancer theranostic applications [16,17]. To illustrate, transition-metal ion copper(II) (Cu(II)), acting as an activator, could induce cancer chemodynamic therapy (CDT). This process facilitates the Cu(II)/Cu(I) conversion, glutathione (GSH) consumption, and high levels of cytotoxic hydroxyl radical (·OH) generation in situ through a Fenton-like reaction involving Cu(I) and endogenous hydrogen peroxide (H_2_O_2_) in the slightly acidic tumor microenvironment (TME). Consequently, this cascade disrupts the redox homeostasis, leads to protein denaturation, and causes DNA damage, ultimately culminating in cancer cell apoptosis [18,19,20]. Furthermore, due to the distinctive reaction conditions of CDT, Cu(II) enables the selective destruction of cancer cells while minimizing damage to surrounding normal tissues, which offers notable advantages over other conventional therapeutic modalities such as chemotherapy and radiotherapy [21,22]. In addition, the lone-pair electrons located in the outer orbital of Cu(II) can interact with neighboring water molecules to shorten the *T*_1_ relaxation time; thus, Cu(II)-based nanocomposites can be employed as *T*_1_-weighted magnetic resonance (MR) imaging contrast agents for tumor diagnosis [23,24]. Despite ongoing developments in Cu(II)-based theranostic applications, some drawbacks persist in Cu(II)-based small-molecular cancer theranostic agents, such as low tumor accumulation and suboptimal spatial and density resolution, significantly impacting the precision and efficiency of tumor theranostics [25,26]. Therefore, it is essential to transport an adequate dosage of Cu(II) to the tumor site for improved accumulation and integrate multiple imaging modalities for improved spatial and density resolution, ultimately facilitating enhanced tumor theranostics.

Over the past decades, a multitude of polymer-based nanocarriers have been developed as promising nanoplatforms that allow the integration of diverse functional modules to address the limitations of small-molecular cancer theranostic agents, such as insufficient tumor accumulation, short circulation half-life, and single functionality [27,28]. Among these polymer-based nanocarriers, branched polyethylenimine (PEI.NH_2_) could afford the loading of imaging elements, targeting agents, drugs, genes, and catalysts simultaneously through its hydrophobic internal cavity and abundant terminal amines, enabling high-efficient diagnostic and therapeutic applications [29,30,31]. In a previous study, Han et al. successfully grafted the bone-targeting agent alendronate (ALN) onto PEI.NH_2_ via linker 4-formylbenzoic acid (CBA), achieving targeted delivery of microRNA and enhancing the efficacy of gene therapy in bone metastatic cancer [32]. Zhu et al. synthesized alkoxyphenyl acylsulfonamide (APAS)-conjugated functionalized PEI.NH_2_-entrapped gold nanoparticles (Au PENPs) with technetium-99m (^99m^Tc) labeling for enhanced single-photon emission computed tomography (SPECT) and computed tomography (CT) imaging of cancer cells. Based on the high X-ray attenuation coefficiency of gold nanoparticles (Au NPs) within the multifunctional PEI.NH_2_, the spatial and density resolution of the dual-mode imaging nanosystem greatly improved, facilitating the precise diagnosis of cancer cells [33]. Li et al. utilized a biomimetic PEI.NH_2_ nanogel system loaded with ultrasmall iron oxide nanoparticles as a vehicle to co-deliver docetaxel and small interfering RNA, enabling MR imaging-guided synergistic chemo/immunotherapy of breast cancer [34]. The above studies fully illustrate that PEI.NH_2_ serves as an ideal nanocarrier, which could incorporate simple modules for effective tumor theranostics.

**Figure 1:**
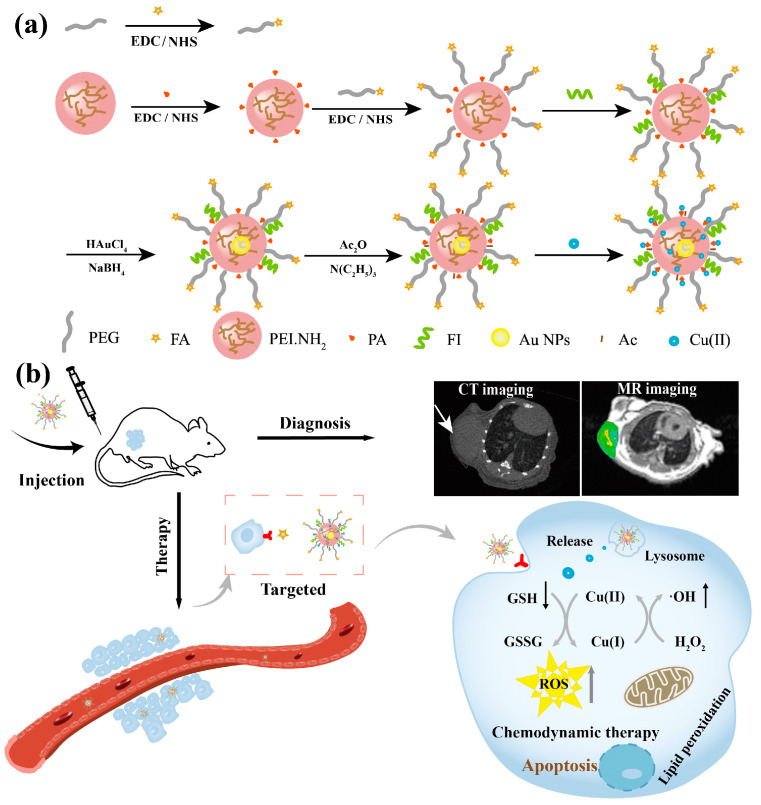
Schematic diagram illustrating (**a**) the synthesis of FA-Au/Cu(II) PENPs for (**b**) targeted tumor CT/MR imaging and chemodynamic therapy.

Given the intrinsic properties of Cu(II) that can simultaneously induce MR imaging and CDT of tumors, as well as PEI.NH_2_ nanotechnology that can improve its tumor accumulation and imaging resolution via multifunctional integration within one entity, we developed folic acid (FA)-linked Cu(II)-based Au PENPs nanocomposites for targeted CT/MR imaging and CDT of tumors. In our work, 2-picolinic acid (PA-COOH), polyethylene glycol (PEG)-linked FA (FA-PEG-COOH), and fluorescein isothiocyanate (FI) were sequentially conjugated with PEI.NH_2_ and then in situ fabrication of Au NPs occurred within the PEI.NH_2_ internal cavity. After acetylation of PEI.NH_2_ terminal amines and Cu(II) complexation, the Cu(II)-based nanocomposites FA-Au/Cu(II) PENPs were generated (Figure 1a). Numerous characterization techniques were utilized to systematically analyze the structure, morphology, and components of FA-Au/Cu(II) PENPs. The stability of colloidal dispersion, the Cu(II) release property, and the Fenton-like reaction process, as well as the FA-mediated targeting specificity, cytotoxicity, and apoptotic mechanism, along with the complementary CT/MR dual-mode imaging and CDT in vitro and in vivo, were systematically studied and analyzed (Figure 1b). To my knowledge, the study demonstrates the first paradigm that integrates Au NPs and Cu(II) into multifunctional PEI.NH_2_ for targeted CT/MR imaging-guided CDT of tumors.

## 2. Materials and Methods

### 2.1. Synthesis of PEI.NH_2_-FI-(PEG-FA)-PA

The functional molecule FA-PEG-COOH (yield = 84.2%) was synthesized through the amidation reaction between the amine group and carboxyl group terminal PEG (NH_2_-PEG-COOH) and FA referring to a specific procedure described in the previous literature [35]. Subsequently, the multifunctional polyethylenimine was synthesized following the preparation procedure depicted in Figure 1a. Briefly, PA-COOH (19.70 mg, 0.160 mmol) dissolved in dimethylformamide (DMF, 10 mL) was first activated by the DMF solution containing 1-ethyl-3-(3-(dimethylamino)propyl) carbodiimide hydrochloride (EDC, 306.72 mg, 1.60 mmol, 25 mL, 10 molar equivalents) and N-hydroxy succinimide (NHS, 184.16 mg, 1.60 mmol, 10 mL, 10 molar equivalents) under intensive magnetic stirring for 3 h. The above-prepared PA-COOH/EDC/NHS mixture was then added dropwise into the 15 mL aqueous solution of PEI.NH_2_ (50.00 mg, 2.00 μmol) following the 80:1 molar ratio between PA-COOH and PEI.NH_2_ and the mixture was continuously stirred for 72 h at room temperature, yielding PEI.NH_2_-PA (yield = 79.5%). Subsequently, using the same EDC/NHS coupling chemistry as above, the prepared 8 mL aqueous solution of FA-PEG-COOH (47.59 mg, 21.52 μmol, 20 molar equivalents) was activated and then reacted with PEI.NH_2_-PA (29.50 mg, 1.076 μmol, 20 mL) solution for 72 h to generate PEI.NH_2_-(PEG-FA)-PA (yield = 86.2%). Finally, 2 mL dimethyl sulfoxide (DMSO) containing FI (1.60 mg, 4.11 μmol, 5 molar equivalents) was reacted with the 15 mL DMSO solution of PEI.NH_2_-(PEG-FA)-PA (50.70 mg, 0.82 μmol) for 24 h in a light-avoiding environment to finish the preparation of PEI.NH_2_-FI-(PEG-FA)-PA (yield = 82.7%). Concurrently, FA-free nanoparticles PEI.NH_2_-FI-mPEG-PA (yield = 78.9%) were synthesized under similar conditions, except that the carboxyl group terminal PEG monomethyl ether (mPEG-COOH) was employed in place of FA-PEG-COOH.

### 2.2. Formation of FA-Au/Cu(II) PENPs

The synthesized PEI.NH_2_-FI-(PEG-FA)-PA was subsequently employed to entrap Au NPs, acetylate the residual amine groups of PEI.NH_2_, and complex with Cu(II), referring to the methods published with slight modifications [36,37,38]. Briefly, 100 molar equivalents of chloroauric acid (HAuCl_4_) solution (0.626 mL, 30 mg/mL, 45.60 μmol) were slowly added into PEI.NH_2_-FI-(PEG-FA)-PA aqueous solution (28.50 mg, 0.456 μmol, 15 mL) and then stirred for 0.5 h. Subsequently, sodium borohydride (NaBH_4_) aqueous solution (5.18 mg, 136.80 μmol, 1 mL) was rapidly added into the above reaction mixture at a 3:1 molar ratio between NaBH_4_ and HAuCl_4_, and the solution was stirred continuously for 2 h to generate the [(Au^0^)_100_-PEI.NH_2_-FI-(PEG-FA)-PA] NPs. Thereafter, triethylamine (30.78 μL, 218.89 μmol) and acetic anhydride (20.90 μL, 218.89 μmol) with 3 times the molar excess of the surface amines of PEI.NH_2_ were utilized to attain acetylation via an additional 24 h amidation reaction. The acquired [(Au^0^)_100_-PEI.NHAc-FI-(PEG-FA)-PA] NPs (denoted as, FA-Au PENPs) aqueous solution was subject to dialysis using a dialysis membrane with a molecular weight cut-off (MWCO) of 8000–14,000 for 3 days to remove the excess reactants or by-products, followed by lyophilization to obtain FA-Au PENPs powder (yield = 88.5%). Finally, FA-Au PENPs (15.00 mg, 0.175 μmol) dispersed in water (20 mL) were mixed with 1 mL cupric chloride (CuCl_2_) aqueous solution (1.80 mg, 10.50 μmol) at an optimal Cu(II) concentration (60 molar equivalents) under ultrasonic stirring for 10 min. After the freeze-drying process, FA-Au/Cu(II) PENPs were obtained (yield = 91.9%). Concurrently, FA-free nanocomposites Au/Cu(II) PENPs (yield = 88.6%) were synthesized using PEI.NH_2_-FI-mPEG-PA as a nanoplatform under similar conditions to facilitate further targeted comparative analysis.

### 2.3. Cu(II) Release Property In Vitro

Triplicate samples of FA-Au/Cu(II) PENPs (1 mg) were individually dispersed in 1 mL of phosphate-citrate buffer with different pH values (5.0, 6.5, and 7.4). Each FA-Au/Cu(II) PENPs dispersion was then sealed in dialysis bags (MWCO = 8000–14,000) and placed in 20 mL of the corresponding phosphate-citrate buffer with the specific pH. The release devices were subsequently put in a vapor-bathing vibrator that maintained a constant temperature of 37 °C. At designated time intervals, 1 mL of the sample from the outer phase buffer medium was collected and supplemented with an equivalent volume of the phosphate-citrate buffer with the corresponding pH. Following this, the Cu(II) concentration in the collected samples was measured by Leeman Prodigy inductively coupled plasma-optical emission spectroscopy (ICP-OES, Hudson, NH, USA) after digestion with aqua regia.

### 2.4. FA-Au/Cu(II) PENPs-Mediated GSH Depletion

The consumption of GSH induced by the released Cu(II) from FA-Au/Cu(II) PENPs was detected using 5,5′-dithiobis-(2-nitrobenzoic acid) (DTNB) as an indicator under different pH conditions according to the reported literature [39,40]. In detail, triplicate FA-Au/Cu(II) PENPs (100 μL, 50 μg/mL) were individually dispersed in PBS solution with different pH values (5.0, 6.5, and 7.4). Each of these solutions was then added to 20 μL of PBS solution containing GSH (0.6 mM) with the corresponding pH and allowed to react for 0.5 h. Subsequently, sodium hydroxide (200 μL, 0.15 mol/L) and formaldehyde (80 μL, 3%) solutions were added to the mixture and incubated for 2 min. DTNB (10 μL, 0.3 mg/mL) dissolved in methanol was added to the above mixture and incubated for an additional 3 min. Finally, the FA-Au/Cu(II) PENPs-mediated GSH depletion was determined by analyzing the mixture’s absorbance at 412 nm using UV-vis spectroscopy (Perkin Elmer, Waltham, MA, USA).

### 2.5. Generation of ·OH by Fenton-like Reaction

The methylene blue (MB) degradation measurements were performed referring to the previous literature [37]. Firstly, the requisite solutions such as FA-Au/Cu(II) PENPs ([Cu] = 10 mM), CuCl_2_ (10 mM), GSH (10 mM), MB (10 μg/mL), and H_2_O_2_ (10 mM) were individually prepared. These solutions with specific concentrations were then mixed in equal volumes according to the following groups: FA-Au/Cu(II) PENPs + MB + GSH + H_2_O_2_, FA-Au/Cu(II) PENPs + MB, CuCl_2_ + MB + GSH + H_2_O_2_, and CuCl_2_ + MB. The MB degradation of the aforementioned solutions was analyzed 2 h later by recording the absorbance at 665 nm using UV-vis spectroscopy (Perkin Elmer, Waltham, MA, USA). Meanwhile, the MB degradation levels of FA-Au/Cu(II) PENPs + MB + GSH + H_2_O_2_ at different time points (0, 30, 60, 90, and 120 min) were also recorded through UV-vis spectroscopy (Perkin Elmer, Waltham, MA, USA).

To assess the effect of pH environments on MB degradation of FA-Au/Cu(II) PENPs + MB + GSH + H_2_O_2_, we recorded the MB degradation over time under different pH conditions. Firstly, MB (10 µg/mL), H_2_O_2_ (10 mM), GSH (10 mM), and FA-Au/Cu(II) PENPs ([Cu] = 10 mM) were separately dissolved in PBS solution with different pH values (5.0, 6.5, and 7.4). Subsequently, equal amounts of FA-Au/Cu(II) PENPs, GSH, H_2_O_2_, and MB solution were thoroughly mixed at room temperature for each pH condition. After that, the absorbance of the mixtures at 665 nm was measured at various time points through UV-vis spectroscopy (Perkin Elmer, Waltham, MA, USA), and the MB residual percentage for each group was calculated statistically.

### 2.6. Targeted CT/MR Imaging In Vitro

4T1 cells (2 × 10^6^ cells/well) were seeded into a 6-well plate and cultured overnight in a 5% CO_2_ incubator maintained at a constant temperature of 37 °C. As for the CT imaging, the cells were treated with fresh medium containing different Au concentrations (0, 25, 50, 100, and 200 µM) of FA-Au/Cu(II) PENPs or Au/Cu(II) PENPs for 2 h. Later, the cells were rinsed, trypsinized, centrifuged, resuspended and transferred into 2 mL centrifuge tubes, and then scanned by a clinical Micro-CT imaging system (SkyScan 1176, Bruker, Berlin, Germany). An identical cell culture protocol was employed for MR imaging. After treatment with FA-Au/Cu(II) PENPs or Au/Cu(II) PENPs with various Cu(II) concentrations (0, 25, 50, 100, and 150 µM) for 2 h, the cells were finally collected and scanned by a 3.0-T MR system (Signa HDxt, GE Medical Systems, Milwaukee, WI, USA).

### 2.7. Cytotoxicity Assay and Cell Morphology Analysis

The cell counting kit-8 (CCK-8, 7Sea Biotech Co., Ltd., Shanghai, China) assay was used to evaluate the inhibition efficiency of the Cu(II)-based nanocomposites towards 4T1 cells. First, 4T1 cells were seeded into a 96-well plate at a density of 1.0 × 10^4^ cells/well. After incubating overnight, the medium was discarded and replaced with 100 μL of fresh medium containing different concentrations of prepared materials (Au PENPs, FA-Au PENPs, CuCl_2_, Au/Cu(II) PENPs, or FA-Au/Cu(II) PENPs), respectively. Then, 24 h later, the medium in each well was replaced with serum-free medium (100 μL) containing CCK-8 solution (10 μL) and the cells were incubated for a further 4 h. The cell culture plate was then placed in a Multiskan MK3 ELISA reader (Thermo Scientific, Waltham, MA, USA), the absorbance at the detected wavelength (450 nm) was recorded, and the cell viability and the half-maximal inhibitory concentration (IC_50_) were calculated. To further investigate the cytotoxicity of nanocomposites towards normal cell lines, CCK-8 assay was performed on normal mouse fibroblast L929 cells via the same method under similar conditions.

The morphological characteristics of 4T1 cells were investigated using an inverted microscope (DM IL LED, Leica, Wetzlar, Germany) after treatment with FA-Au/Cu(II) PENPs, Au/Cu(II) PENPs, and CuCl_2_ at the same Cu(II) concentration ([Cu] = 1000 μM), as well as with FA-Au PENPs and Au PENPs at a comparable carrier concentration ([PEI.NH_2_] = 16 μM), which corresponded to the carrier concentration of FA-Au/Cu(II) PENPs and Au/Cu(II) PENPs above.

### 2.8. Cell Apoptosis Assay

The apoptosis level of 4T1 cells with treatment of FA-Au/Cu(II) PENPs was further studied through a cell sorting method by flow cytometry using Annexin V-labeled fluorescein isothiocyanate (V-FITC)/propidium iodide (PI) Apoptosis Kit (Beyotime Biotechnology, Shanghai, China) following the standard protocol of the manufacturer. To eliminate potential fluorescence interference from FI, FA-Au/Cu(II) PENPs without FI conjugation were prepared for cell apoptosis evaluation in this experiment. First, 4T1 cells (2 × 10^5^ cells/well) were seeded in 12-well plates and cultured overnight. After the cell adherent growth, the medium was replaced with the medium containing FA-Au/Cu(II) PENPs or CuCl_2_ ([Cu] = 10 or 100 μM) and incubated for 24 h. 4T1 cells with treatment of PBS were set as the control. Following this, the cells in each well were trypsinized, washed, and collected. Subsequently, the cells in each well were resuspended in 195 μL of pre-cooled binding solution, then stained with Annexin V-FITC (5 μL) and PI (10 μL) in a light-avoiding environment for 15 min. Finally, 20,000 cells from each sample were counted and sorted using a FACScan analyzer (Becton Dickinson, Franklin, CA, USA) for cell apoptosis analysis.

### 2.9. Determination of Intracellular ROS and LPO Levels

To determine the intracellular reactive oxygen species (ROS) levels of 4T1 cells with treatment of FA-Au/Cu(II) PENPs, 2′,7′-dichlorofluorescin diacetate (DCFH-DA, Beyotime Biotechnology, Shanghai, China) was utilized as an oxidative-responsive fluorescent probe to test ROS generation. Consistent with the methodology used in the Annexin V-FITC/PI apoptosis assay, FA-Au/Cu(II) PENPs without FI conjugation were utilized here. Firstly, 4T1 cells (2 × 10^5^ cells/well) were seeded in a 6-well plate and cultured for adherent growth. Subsequently, each well of 4T1 cells was separately supplemented with medium containing FA-Au/Cu(II) PENPs or CuCl_2_ with different Cu(II) concentrations (10 and 100 μM) and incubated for an additional 5 h. 4T1 cells with treatment of PBS were used as the control. Following this, 4T1 cells were washed and then incubated with 1 mL of serum-free medium containing DCFH-DA (10 μM) for 40 min in a light-avoiding environment. Ultimately, the cell sample from each well was washed and detected using a FACScan analyzer (Becton Dickinson, Franklin, CA, USA). In terms of intracellular lipid peroxidation (LPO) analysis, the same cell culture procedures were performed, except that the oxidative-sensitive fluorescent probe C11-BODIPY^581/591^ (GlpBio Technology, Montclair, CA, USA) was utilized in place of DCFH-DA.

### 2.10. Targeted CT/MR Imaging In Vivo

Animal experiments were conducted in adherence to the standard protocols approved by the Animal Ethics Committee of Nanjing Tech University (ethical certificate number: IACUC-20240310-12). Female nude mice (BALB/c-nu, 4–5 weeks, 18–22 g) were sourced from Shanghai Slac Laboratory Animal Center (Shanghai, China). Xenograft models were initially established in BALB/c-nu mice by subcutaneously injecting approximately 5 × 10^6^ 4T1 cells/mouse into their right upper limbs. Upon reaching a tumor volume within the range of 0.5–1.0 cm^3^, the mice were randomly allocated into two experimental groups for the subsequent in vivo CT/MR imaging studies, in accordance with the reported literature [36,38,41]. Before the imaging scan, each 4T1-tumor-bearing mouse was anesthetized and subsequently administered with 100 μL of normal saline (NS) containing FA-Au/Cu(II) PENPs or Au/Cu(II) PENPs via tail vein injection. For CT imaging, FA-Au/Cu(II) PENPs and Au/Cu(II) PENPs solutions with a Au concentration of 0.08 M were employed. As for MR imaging, FA-Au/Cu(II) PENPs and Au/Cu(II) PENPs solutions with a Cu(II) concentration of 8 mM were utilized. After the administration, CT and MR images of the 4T1-tumor-bearing mice were captured at various time points using a clinical Micro-CT imaging system (SkyScan 1176, Bruker, Berlin, Germany) and a 3.0-T MR system (Signa HDxt, GE Medical Systems, Milwaukee, WI, USA), respectively. The instrumental parameters were set referring to the literature [36,41]. The Hounsfield units (HUs) from CT imaging and the signal-to-noise-ratios (SNRs) from MR imaging of tumor sites at corresponding time points were recorded and analyzed ultimately.

### 2.11. CDT of Tumor In Vivo

Following the successful establishment of the xenograft models (designated as day 1), the mice were randomly assigned to five experimental groups (4 4T1-tumor-bearing mice/group), including NS, FA-Au PENPs, Au PENPs, FA-Au/Cu(II) PENPs, and Au/Cu(II) PENPs. Subsequently, 100 μL of NS, FA-Au/Cu(II) PENPs ([Cu] = 8 mM), Au/Cu(II) PENPs ([Cu] = 8 mM), and FA-Au PENPs with the same carrier concentration of FA-Au/Cu(II) PENPs ([Cu] = 8 mM), and Au PENPs with the same carrier concentration of Au/Cu(II) PENPs ([Cu] = 8 mM), were intravenously administered to the mice of the corresponding groups, respectively. Treatment was initiated on day 1 and subsequently administered every 3 days for a total of 6 doses. Throughout the experimental period, body weight (W_t_), survival rate, and tumor volume (V_t_) of each 4T1-tumor-bearing mouse in every mice group were recorded to evaluate the biosafety and CDT efficiency of the Cu(II)-based nanocomposites following the methods published in the literature [35,38]. Upon completion of the administration, tumors and major organs were dissected and collected from a euthanized mouse in each mice group. Through fixing, dehydration, embedding, slicing, and staining with hematoxylin–eosin (H&E), the acquired tissue sections of major organs and tumors were observed to analyze the levels of cell necrosis. In addition, a terminal deoxynucleotidyl transferase dUTP nick end labeling (TUNEL) assay was utilized to locate the apoptotic areas and determine the apoptosis rate of the ex vivo tumors. The acquisition and staining procedures of the pathological sections were similar to the standard protocols published in previous studies [36,38,41]. See full experimental details in the Supporting Information.

## 3. Results and Discussion

### 3.1. Preparation and Characterization of FA-Au/Cu(II) PENPs

To deliver a sufficient dosage of Cu(II) to the tumor site and integrate multiple imaging modalities to achieve precise and effective cancer theranostics, the Cu(II)-based nanocomposites FA-Au/Cu(II) PENPs were synthesized following the procedures outlined in Figure 1. PEI.NH_2_ was first linked to PA-COOH to enhance the Cu(II) complexing efficiency. This was followed by sequential conjugation with functional moieties (PEGylated FA and FI) and in situ fabrication of Au NPs within the PEI.NH_2_ internal cavity. Subsequently, through acetylation and Cu(II) complexation, the Cu(II)-based nanocomposites FA-Au/Cu(II) PENPs were generated and comprehensively characterized.

To confirm the successful synthesis of intermediate chemicals (FA-PEG-COOH, PEI.NH_2_-PA, PEI.NH_2_-(PEG-FA)-PA, PEI.NH_2_-FI-(PEG-FA)-PA, PEI.NH_2_-mPEG-PA, and PEI.NH_2_-FI-mPEG-PA), proton nuclear magnetic resonance (^1^H NMR) spectroscopy was utilized. Via qualitative analysis and quantitative calculation of a series of functional moieties, approximately 23.0 PA-COOH, 15.6 FA-PEG-COOH, 7.8 FA, and 2.2 FI were determined in the PEI.NH_2_-FI-(PEG-FA)-PA compound. The comparable functional moieties (15.8 mPEG-COOH and 2.0 FI) were quantitatively determined in the PEI.NH_2_-FI-mPEG-PA compound (Figure S1). The UV-vis spectroscopy results further verified the formation of PEI.NH_2_-FI-(PEG-FA)-PA based on the coexistence of characteristic peaks of PA-COOH (265 nm), FA (278 nm and 360 nm), and FI (511 nm) in the UV-vis spectrum of PEI.NH_2_-FI-(PEG-FA)-PA (Figure S2).

Subsequently, a series of UV-vis spectroscopic characterizations were conducted to determine the Cu(II) complexing capacity of the multifunctional polyethylenimine nanoplatform according to the method used in the previous literature [42]. Through comprehensive analysis, the optimal complexing equivalent of Cu(II) was determined to be 60 in both the PEI.NHAc-FI-(PEG-FA)-PA/Cu(II) complexes and PEI.NHAc-FI-mPEG-PA/Cu(II) complexes (Figure S3). Obviously, the optimal complexing equivalent of Cu(II) in PEI.NHAc-FI-(PEG-FA)-PA/Cu(II) complexes is much higher than the number of PA-COOH moieties in these complexes, probably due to the abundant secondary and tertiary amine complexing sites in the multifunctional polyethylenimine skeleton that enable additional Cu(II) complexation.

Furthermore, through comparing the UV-vis spectra of CuCl_2_, PEI.NHAc-FI-(PEG-FA)-PA, and PEI.NHAc-FI-(PEG-FA)-PA/60Cu(II) complexes, it can be easily observed that the absorption peak of CuCl_2_ (814 nm) is blue-shifted to 656 nm in the PEI.NHAc-FI-(PEG-FA)-PA/60Cu(II) complexes, indicating that the change in complexing environment leads to a change in the UV-vis absorption peak of Cu(II)-based complexes. Additionally, there is no characteristic absorption peak of free Cu(II) (814 nm) in the UV-vis spectrum of PEI.NHAc-FI-(PEG-FA)-PA/60Cu(II) complexes, which suggests that Cu(II) is fully complexed within PEI.NHAc-FI-(PEG-FA)-PA (Figure S4). Consequently, the polyethylenimine derivatives were employed to complex 60 molar equivalents of Cu(II) per PEI.NH_2_ for subsequent studies, ensuring no leakage of excess Cu(II).

**Figure 2:**
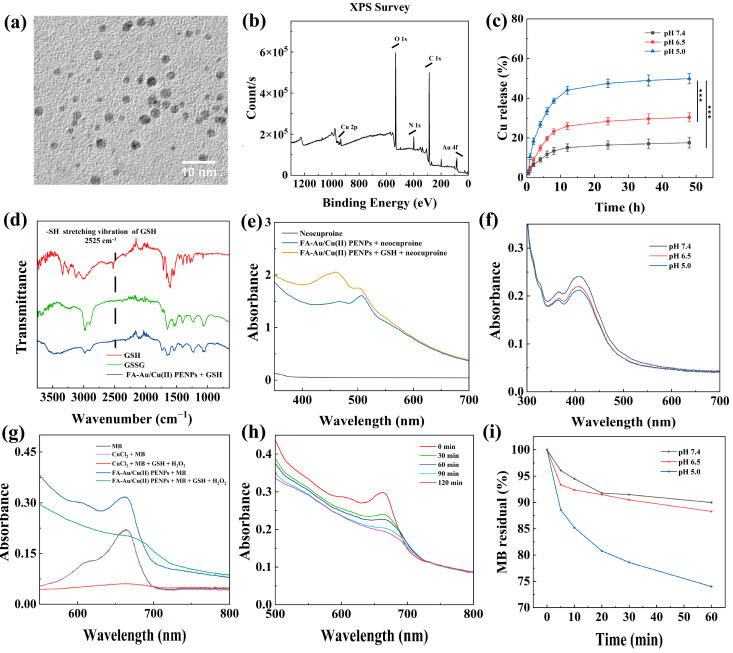
(**a**) TEM image and (**b**) XPS survey spectrum of the FA-Au/Cu(II) PENPs. (**c**) Release of Cu(II) from FA-Au/Cu(II) PENPs under different pH conditions. (**d**) FT-IR spectra of GSH, GSSG, and FA-Au/Cu(II) PENPs + GSH. (**e**) UV-vis spectra of the solution of FA-Au/Cu(II) PENPs mixed with or without GSH and reacted with neocuproine. (**f**) UV-vis spectra of DTNB presented GSH depletion by FA-Au/Cu(II) PENPs under different pH conditions. (**g**) UV-vis spectra of CuCl_2_, FA-Au/Cu(II) PENPs, CuCl_2_ + GSH + H_2_O_2_, and FA-Au/Cu(II) PENPs + GSH + H_2_O_2_ treated with MB for 2 h, respectively. (**h**) The degradation curves of MB over time after mixing with FA-Au/Cu(II) PENPs + GSH + H_2_O_2_. (**i**) Percentage of MB residual in FA-Au/Cu(II) PENPs + GSH + H_2_O_2_ + MB over time under different pH conditions (*** *p* < 0.001).

Following the in situ fabrication of Au NPs within the polyethylenimine derivative internal cavity and the complexation of Cu(II) at an optimal molar equivalents, the FA-Au/Cu(II) PENPs with excellent water solubility were formed. The prominent surface plasmon resonance (SPR) peak of Au NPs, located at 502 nm, emerges in the UV-vis spectrum of FA-Au/Cu(II) PENPs, attesting to the successful synthesis of Au NPs (Figure S5). The result is consistent with the findings of a previous study [33]. As seen through transmission electron microscopy (TEM) observation, FA-Au/Cu(II) PENPs exhibit a spherical morphology with a uniform distribution of particle sizes (2.87 ± 0.52 nm, Figure 2a and Figure S6a). Under high-resolution TEM, FA-Au/Cu(II) PENPs show a distinct two-dimensional lattice structure (Figure S6b). Furthermore, four distinct characteristic rings (111), (200), (220), and (311) can be observed in the selected-area electron diffraction (SAED) patterns, indicating that the synthesized FA-Au/Cu(II) PENPs have a face-centered cubic crystal structure (Figure S6c). By employing X-ray photoelectron spectroscopy (XPS) for the characterization of FA-Au/Cu(II) PENPs, both the element species and copper valence state can be accurately determined. After being calibrated by the C 1s peak (284.9 eV), the presence of O, N, C, Au, and Cu elements within the FA-Au/Cu(II) PENPs can be confirmed (Figure 2b). This finding verifies the successful entrapment of Au NPs and the complexation of Cu(II) in FA-Au/Cu(II) PENPs. As further illustrated in Figure S7, the Cu 2P_1/2_ and Cu 2P_3/2_ peaks were detected at 953.2 eV and 933.4 eV, respectively, accompanied by a satellite peak at 943.1 eV. These spectral features indicate the presence of Cu(II) in the FA-Au/Cu(II) PENPs, which is identical to the literature [43]. To determine the extra complexation mode of Cu(II) in FA-Au PENPs, Fourier-transform infrared (FT-IR) spectroscopy was utilized. As revealed in Figure S8, the intensity of the bending vibration peak corresponding to secondary amines (1550–1660 cm^−1^) in FA-Au/Cu(II) PENPs exhibits a significant decrease in comparison to that of FA-Au PENPs, indicating that Cu(II) could complex with FA-Au PENPs through secondary amines in the multifunctional polyethylenimine skeleton, in keeping with a previous study [44]. Finally, the weight percentages of Cu(II) and Au in the FA-Au/Cu(II) PENPs can be determined to be 3.98% and 20.58%, respectively, through ICP-OES measurements. After determining all of the Cu(II) complexing sites on the functionalized polyethylenimine, it can be concluded that Au NPs and Cu(II) do not interact with each other in terms of spatial location and reactive sites, because Au NPs are in situ fabricated with the spherical morphology within the internal cavity of the functionalized polyethylenimine. Although Au NPs occupy a certain space within the internal cavity of branched polyethylenimine, Cu(II) possesses many more complexing sites on the surface (PA-COOH moieties) and the internal skeleton (secondary amines) of the functionalized polyethylenimine, which are hardly affected by Au NPs.

The hydrodynamic sizes and zeta potentials of FA-Au/Cu(II) PENPs and FA-Au PENPs were further measured. It is noteworthy that the hydrodynamic size of FA-Au/Cu(II) PENPs as measured by dynamic light scattering (DLS) is 222.2 ± 21.7 nm, which is smaller than that of FA-Au PENPs (252.2 ± 8.5 nm, Table S1 and Figure S9). This phenomenon is likely attributable to the extra complexation between Cu(II) and secondary amines in the multifunctional polyethylenimine skeleton, leading to the contraction of the functionalized polyethylenimine derivatives and a modest decrease in the hydrodynamic size of FA-Au/Cu(II) PENPs, in keeping with a previous study [38]. Moreover, after Cu(II) complexation, the zeta potential of FA-Au/Cu(II) PENPs (2.5 ± 2.0 mV) increases slightly in comparison to that of FA-Au PENPs (−14.2 ± 0.3 mV), which is identical to the previous study (Table S1) [37]. Interestingly, the hydrodynamic size of FA-Au/Cu(II) PENPs (222.2 ± 21.7 nm) determined by DLS exceeds the diameter of FA-Au/Cu(II) PENPs (2.87 ± 0.52 nm) obtained by TEM. This discrepancy can be attributed to the fact that DLS measures the nanoclusters’ hydrodynamic size of FA-Au/Cu(II) PENPs in aqueous solution which consists of numerous single FA-Au/Cu(II) PENPs, whereas TEM tests the size of individual Au core NPs in a dried state, in keeping with a previous study [45]. In the aqueous solution of FA-Au/Cu(II) PENPs, the hydrogen bonding interactions among functionalized polyethylenimines likely result in mutual adsorption. Furthermore, solvent molecules can be trapped between functionalized polyethylenimines, thus forming the aggregation. Ultimately, the aggregation behavior of these nanoparticles induces the formation of nanoclusters, which demonstrates the relatively large hydrodynamic size measured by DLS, in agreement with a previous study [46].

### 3.2. In Vitro Stability and Hemocompatibility Assessments

As a crucial prerequisite for further in vivo biomedical applications, it is necessary to investigate the colloidal stability of FA-Au/Cu(II) PENPs via UV-vis spectroscopy and DLS measurements. As anticipated and illustrated in Figure S10, the uniform SPR peaks without notable alteration and shift are demonstrated in the UV-vis spectra of FA-Au/Cu(II) PENPs after being subjected to various temperatures (4–50 °C) and storage time (1–7 days). Furthermore, whether dispersed in water or PBS, the hydrodynamic sizes, polydispersity indexes (PDIs), and zeta potentials of FA-Au/Cu(II) PENPs barely change within 7 days (Figure S11 and Table S2). These findings indicate that FA-Au/Cu(II) PENPs possess favorable stability of colloidal dispersion to maintain uniformity.

To ensure the subsequent safe application of Cu(II)-based nanocomposites in vivo, the hemocompatibility of FA-Au/Cu(II) PENPs was assessed by a hemolytic assay. As depicted in Figure S12, the hemolysis rates of red blood cells with treatment of FA-Au/Cu(II) PENPs at different Cu concentrations (0–40 μg/mL) are less than 5%. Furthermore, these treated red blood cells maintain their integrity when compared to the ruptured cells with treatment of water (positive control) in the inserted photograph of Figure S12. The satisfactory hemocompatibility result implies that FA-Au/Cu(II) PENPs are suitable candidates for subsequent biomedical applications.

### 3.3. Cu(II) Release Study In Vitro

To study the release of Cu(II) from FA-Au/Cu(II) PENPs in various physiological environments including plasma, tumor, and lysosome, FA-Au/Cu(II) PENPs were exposed to buffers with different pH values (7.4, 6.5, and 5.0) for simulation. As illustrated in Figure 2c, the accumulative release percentages of Cu(II) from FA-Au/Cu(II) PENPs in different pH buffers reach a plateau after 12 h and attain their maximum at 48 h. At 48 h, 49.9% of Cu(II) is released from FA-Au/Cu(II) PENPs in the pH 5.0 buffer, demonstrating the significantly higher release amount of Cu(II) than that in the pH 6.5 (30.4%) and pH 7.4 (17.6%) buffers (*p* < 0.001). It can be concluded that the decrease in pH leads to an increase in the release amount of Cu(II) from FA-Au/Cu(II) PENPs. The pH-dependent Cu(II) release property of FA-Au/Cu(II) PENPs can be attributed to the competitive binding of protons with secondary amines of the multifunctional polyethylenimine skeleton. Under lower pH conditions, the higher degree of protonation of secondary amines of the multifunctional polyethylenimine skeleton probably weakens the complexation between Cu(II) and secondary amines of the multifunctional polyethylenimine skeleton, facilitating the higher amount of Cu(II) release from the complexing sites of FA-Au/Cu(II) PENPs, in agreement with a previous study [47].

### 3.4. FA-Au/Cu(II) PENPs-Mediated GSH Depletion and ·OH Generation

Recent studies have elucidated the mechanism of Cu(II)-mediated tumor CDT in the environment with excessive GSH and endogenous H_2_O_2_. This CDT mechanism primarily encompasses two processes, as follows. Initially, Cu(II) consumes intracellular GSH to generate oxidized GSH (GSSG) and Cu(I). Subsequently, the generated Cu(I) reacts with endogenous H_2_O_2_ to produce ·OH via a Fenton-like reaction, ultimately inducing the apoptosis of cancer cells [22,48,49].

To verify that FA-Au/Cu(II) PENPs could achieve the first process of the mechanism, FT-IR and UV-vis spectroscopic characterizations were utilized. Through comparing the FT-IR spectra of GSH, GSSG, and the reaction mixture of FA-Au/Cu(II) PENPs and GSH, the stretching vibration peak (2525 cm^−1^) ascribed to the sulfhydryl group (-SH) of GSH disappeared in the FT-IR spectrum of FA-Au/Cu(II) PENPs + GSH, indicating the transformation from GSH to GSSG due to the FA-Au/Cu(II) PENPs’ mediation (Figure 2d). To further confirm the generation of Cu(I) resulting from the reduction of Cu(II), neocuproine can be employed as an indicator due to its exhibition of a characteristic absorption peak (461 nm) in UV-vis spectroscopy upon reacting with Cu(I). As illustrated in Figure 2e, the mixture of FA-Au/Cu(II) PENPs and neocuproine exhibits the characteristic absorption peak located at 461 nm only in the presence of GSH, suggesting that the Cu(II) of FA-Au/Cu(II) PENPs can be reduced to Cu(I) by GSH. Furthermore, based on the principle that DTNB could react with GSH to produce 2-nitro-5-mercaptobenzoic acid (TNB), which exhibits a distinct absorption peak (412 nm) in UV-vis spectroscopy, DTNB can be employed as an indicator to detect GSH depletion. After the 0.5 h reaction with FA-Au/Cu(II) PENPs under different pH conditions (5.0, 6.5, and 7.4), the residual GSH was determined by DTNB through analyzing the mixture’s absorbance at 412 nm. As revealed in Figure 2f, the absorbance at 412 nm decreases with the decrease in pH, suggesting that FA-Au/Cu(II) PENPs could release more Cu(II) to consume GSH at a lower pH.

To further verify the second process of the mechanism, MB degradation measurements were performed, evaluating the ability of FA-Au/Cu(II) PENPs to generate ·OH via a Fenton-like reaction. As illustrated in Figure 2g, the absorbance of MB at 665 nm did not decrease when MB was mixed only with FA-Au/Cu(II) PENPs or CuCl_2_ in the absence of GSH and H_2_O_2_. However, the absorbance of MB at 665 nm decreased significantly when MB was mixed with FA-Au/Cu(II) PENPs or CuCl_2_ in the presence of GSH and H_2_O_2_. This reveals that FA-Au/Cu(II) PENPs could effectively generate ·OH via a Fenton-like reaction in the presence of GSH and H_2_O_2_. And with the treatment of FA-Au/Cu(II) PENPs in the presence of GSH and H_2_O_2_, the absorbance of MB at 665 nm displays a decreasing trend over time (Figure 2h). This reveals that FA-Au/Cu(II) PENPs could continuously generate ·OH in a time-dependent manner. Moreover, the investigation reveals that the residual MB levels of the mixture of FA-Au/Cu(II) PENPs and MB in the presence of GSH and H_2_O_2_ significantly decrease with the decrease in pH (from 7.4 to 5.0), suggesting that an acidic environment enhances the production of ·OH by FA-Au/Cu(II) PENPs, thereby promoting MB degradation (Figure 2i). Overall, these results confirm that FA-Au/Cu(II) PENPs could efficiently generate ·OH in the presence of GSH and H_2_O_2_, particularly under slightly acidic conditions.

### 3.5. Targeting Specificity and CT/MR Imaging In Vitro

**Figure 3:**
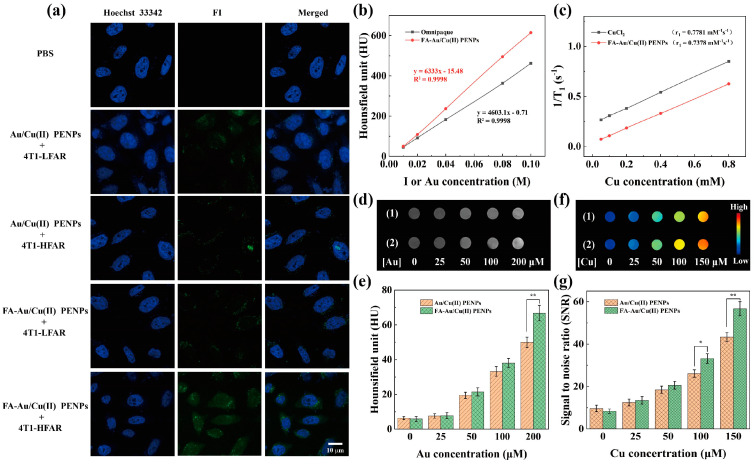
(**a**) Confocal microscopy images of 4T1-LFAR and 4T1-HFAR cells treated with PBS, Au/Cu(II) PENPs, and FA-Au/Cu(II) PENPs for 2 h, respectively. The scale bar represents 10 μm. The linear fitting of (**b**) X-ray attenuation intensity versus radiodense element concentration of FA-Au/Cu(II) PENPs and Omnipaque, respectively, and (**c**) 1/*T*_1_ versus Cu concentration of FA-Au/Cu(II) PENPs and CuCl_2_, respectively. (**d**) CT images and (**e**) the corresponding CT values of 4T1 cells with treatment of (1) Au/Cu(II) PENPs and (2) FA-Au/Cu(II) PENPs at different Au concentrations for 2 h, respectively. (**f**) *T*_1_-weighted MR pseudo-color images and (**g**) the corresponding MR values of 4T1 cells with treatment of (1) Au/Cu(II) PENPs and (2) FA-Au/Cu(II) PENPs at different Cu concentrations for 2 h, respectively (* *p* < 0.05 and ** *p* < 0.01).

To confirm the expected targeting specificity of FA-Au/Cu(II) PENPs due to the FA conjugation, flow cytometry, confocal microscopy, and ICP-OES were employed to determine the cellular uptake of FA-Au/Cu(II) PENPs. Firstly, based on the previous literature, 4T1 cells were pre-incubated with or without FA to obtain two specific cell lines, respectively: 4T1 cells with low-level FA receptor expression (4T1-LFAR) and 4T1 cells with high-level FA receptor expression (4T1-HFAR) [50]. Flow cytometric results reveal that 4T1-HFAR cells incubated with FA-Au/Cu(II) PENPs exhibit markedly higher FI-associated fluorescence intensity than that of 4T1-LFAR cells with the same treatment. In contrast, 4T1-LFAR and 4T1-HFAR cells treated with Au/Cu(II) PENPs exhibit relatively low fluorescence intensity (Figure S13). This suggests that the binding specificity between FA-Au/Cu(II) PENPs and 4T1-HFAR cells induces higher cellular uptake via a receptor-mediated targeting mechanism. Likewise, quantitative analysis of the mean fluorescence intensity and cells taken up with FA-Au/Cu(II) PENPs and Au/Cu(II) PENPs corroborates the fluorescent intensity observations, revealing that 4T1-HFAR cells treated with FA-Au/Cu(II) PENPs show significantly higher mean fluorescence intensity and cellular uptake than those of 4T1-LFAR cells (*p* < 0.001, Figure S14). The targeting specificity of FA-Au/Cu(II) PENPs towards 4T1-HFAR cells was further verified using confocal microscopy due to the visual fluorescence of FI on FA-Au/Cu(II) PENPs. 4T1-HFAR cells with treatment of FA-Au/Cu(II) PENPs exhibit the most prominent FI fluorescence signal among all of the cells, indicating that the specific cellular uptake of FA-Au/Cu(II) PENPs occurs in the 4T1-HFAR cells (Figure 3a). Similarly, the targeted cellular uptake of FA-Au/Cu(II) PENPs was determined by ICP-OES through analyzing the Au content in 4T1-LFAR and 4T1-HFAR cells. As revealed in Figure S15, both 4T1-LFAR and 4T1-HFAR cells internalize Au in a concentration-dependent manner. Notably, the cellular uptake of Au in 4T1-HFAR cells is significantly higher than that in 4T1-LFAR cells at the given Au concentrations (50, 100, and 200 μM, *p* < 0.05). Overall, these results jointly confirm that FA-Au/Cu(II) PENPs have the ability to be specifically delivered to FAR-overexpressing cancer cells, facilitating specific cellular uptake.

To figure out the CT/MR imaging performance of FA-Au/Cu(II) PENPs, X-ray attenuation assay and *T*_1_-weighted MR relaxometry studies were performed. Interestingly, FA-Au/Cu(II) PENPs have superior brightnesses in CT imaging and higher CT values than those of Omnipaque at the same radiodense element concentrations, indicating the excellent X-ray attenuation property of FA-Au/Cu(II) PENPs (Figure 3b and Figure S16a). Furthermore, FA-Au/Cu(II) PENPs demonstrate the increased MR signal intensity in a Cu-concentration-dependent manner. Through linear fitting of Cu(II) concentration and the inverse of the longitudinal relaxation time (1/*T*_1_), the r_1_ relaxivity of FA-Au/Cu(II) PENPs was determined to be 0.7378 mM^−1^s^−1^, which is comparable to that of CuCl_2_ (0.7781 mM^−1^s^−1^, Figure 3c and Figure S16b).

In light of the encouraging results, FA-Au/Cu(II) PENPs were subsequently employed for CT/MR imaging in vitro. As illustrated in Figure 3d, the CT image brightness of 4T1 cells increases with the increase in Au concentration for both FA-Au/Cu(II) PENPs- and Au/Cu(II) PENPs-treated cells. Notably, the CT signal value of 4T1 cells incubated with FA-Au/Cu(II) PENPs is higher than that of 4T1 cells incubated with Au/Cu(II) PENPs, particularly at 200 μM of Au concentration (*p* < 0.01, Figure 3e). In terms of MR imaging of cells in vitro, the MR signal intensity of 4T1 cells is positively correlated with Cu concentration for both FA-Au/Cu(II) PENPs- and Au/Cu(II) PENPs-treated cells (Figure 3f). Obviously, the MR SNR value of 4T1 cells cultured with FA-Au/Cu(II) PENPs is higher than that of 4T1 cells treated with Au/Cu(II) PENPs, particularly at the relatively high Cu concentrations (100 and 150 μM, *p* < 0.05, Figure 3g). These results vastly suggest that FA-mediated targeting specificity could enhance the cellular uptake of FA-Au/Cu(II) PENPs, facilitating the targeted CT/MR imaging of cancer cells in vitro.

### 3.6. FA-Au/Cu(II) PENPs-Mediated CDT In Vitro

**Figure 4:**
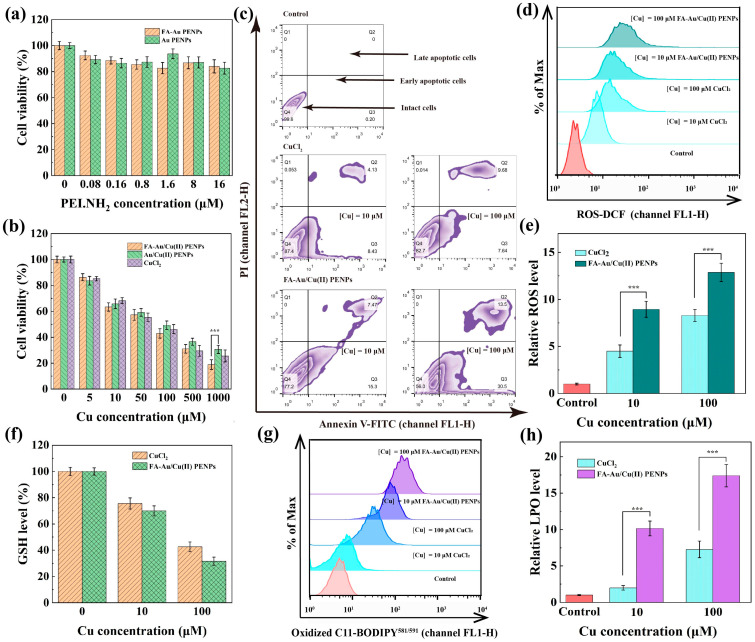
Cell viability of 4T1 cells after 24 h incubation with (**a**) FA-Au PENPs, Au PENPs at different PEI.NH_2_ concentrations, and (**b**) FA-Au/Cu(II) PENPs, Au/Cu(II) PENPs, and CuCl_2_ at different Cu concentrations. (**c**) Flow cytometric profiles measured using Annexin V-FITC/PI double staining for the apoptosis analysis of 4T1 cells after incubation with different Cu concentrations of CuCl_2_ and FA-Au/Cu(II) PENPs for 24 h. The intracellular (**d**) ROS characterization graph, (**e**) the corresponding quantitative analysis of ROS levels, (**f**) GSH levels, (**g**) LPO characterization graph, and (**h**) the corresponding quantitative analysis of LPO levels in the 4T1 cells incubated with CuCl_2_ and FA-Au/Cu(II) PENPs at different Cu concentrations for 5 h (*** *p* < 0.001).

Given the Cu(II)-induced CDT of cancer, it is essential to assess the inhibition efficacy of FA-Au/Cu(II) PENPs on cancer cells in vitro. Firstly, CCK-8 assay was utilized to evaluate the cytotoxicity of FA-Au PENPs and FA-Au/Cu(II) PENPs on 4T1 cells after 24 h incubation. As illustrated in Figure 4a, the viabilities of 4T1 cells incubated with FA-Au PENPs and Au PENPs exceed 82.7% within the given PEI.NH_2_ concentration range (0–16 μM), suggesting their excellent cytocompatibility on 4T1 cells. However, after Cu(II) complexing, Au/Cu(II) PENPs and FA-Au/Cu(II) PENPs display notable inhibition efficacy on 4T1 cells within the given Cu concentration range (0–1000 μM), which correspond to the carrier concentration of Au PENPs and FA-Au PENPs above. And the inhibition efficacies of Au/Cu(II) PENPs and FA-Au/Cu(II) PENPs on 4T1 cells increase with the increase in Cu concentration, which is similar to that of CuCl_2_. It is worth noting that FA-Au/Cu(II) PENPs exhibit much higher cytotoxicity than that of Au/Cu(II) PENPs at the Cu concentration of 1000 μM (*p* < 0.001). This enhanced cytotoxicity is likely attributed to the targeting specificity between FA receptors overexpressed on 4T1 cells and FA, which facilitates increased cellular uptake of FA-Au/Cu(II) PENPs (Figure 4b). Furthermore, the IC_50_ values of FA-Au/Cu(II) PENPs, Au/Cu(II) PENPs, and CuCl_2_ against 4T1 cells after 24 h incubation were calculated and presented in Table S3. The FA-Au/Cu(II) PENPs present the lowest IC_50_ value (70.80 μM) among all the materials recorded here. The enhanced inhibition efficacy of FA-Au/Cu(II) PENPs on 4T1 cells should be attributed to the superior targeting effect compared to Au/Cu(II) PENPs and the higher CDT efficacy than that of the small-molecular therapeutic agent CuCl_2_. Morphological analysis of cells further confirms the enhanced inhibition efficacy of FA-Au/Cu(II) PENPs on 4T1 cells. As revealed in Figure S17, FA-Au/Cu(II) PENPs ([Cu] = 1000 μM)-treated cells showed the largest area of non-adherent and shrunken cells among all of the material-treated cells. Conversely, no detached cells could be observed in the cells treated with Au PENPs and FA-Au PENPs at the corresponding carrier concentration of Au/Cu(II) PENPs and FA-Au/Cu(II) PENPs, respectively, similarly to the PBS-treated cells. Overall, the generated FA-Au/Cu(II) PENPs exhibit desired inhibition efficacy on 4T1 cells, indicating that the bioactivity related to anti-cancer efficacy is primarily associated with the complexed Cu(II). However, FA-Au/Cu(II) PENPs, Au/Cu(II) PENPs, and CuCl_2_ exhibit negligible cytotoxicity towards normal mouse fibroblast L929 cells within the given Cu concentration range (0–1000 μM), as well as Au PENPs and FA-Au PENPs within the given PEI.NH_2_ concentration range (0–16 μM, Figure S18). The results indicate that FA-Au/Cu(II) PENPs and Au/Cu(II) PENPs could selectively inhibit tumor cells with high levels of endogenous H_2_O_2_, displaying no obvious inhibitory effect on normal cells.

To verify the competitive binding effect of FA to FA receptors on the surface of 4T1 cells, the targeted cancer cell inhibition of FA-Au/Cu(II) PENPs was performed using CCK-8 assay. As illustrated in Figure S19, 4T1-HFAR cells treated with FA-Au/Cu(II) PENPs exhibit significantly lower cell viability (64.4%) than that of 4T1-HFAR cells incubated with Au/Cu(II) PENPs and 4T1-LFAR cells incubated with Au/Cu(II) PENPs or FA-Au/Cu(II) PENPs (*p* < 0.001). This indicates that the competitive binding of FA to FA receptors on the surface of 4T1 cells results in the decreased cellular uptake of FA-Au/Cu(II) PENPs by 4T1-LFAR cells, ultimately leading to the relatively low cancer cell inhibition of FA-Au/Cu(II) PENPs on 4T1-LFAR cells. Furthermore, the FA-mediated targeting specificity between FA-Au/Cu(II) PENPs and 4T1-HFAR cells enables a targeted inhibitory effect on 4T1-HFAR cells through targeted cellular uptake.

To deeply evaluate the apoptotic efficacy of 4T1 cells after incubation with CuCl_2_ and FA-Au/Cu(II) PENPs, the Annexin V-FITC/PI Apoptosis Kit was utilized. As illustrated in Figure 4c, the treatments of FA-Au/Cu(II) PENPs and CuCl_2_ for 24 h at the given concentrations ([Cu] = 10 and 100 μM) led to reduced intact cells and increased early apoptotic and late apoptotic cells than that of PBS. Through further analyzing the percentage of 4T1 cells classified into different stages, the apoptotic efficiencies of CuCl_2_ and FA-Au/Cu(II) PENPs could be determined. Notably, FA-Au/Cu(II) PENPs induced significantly less intact cells and more early apoptotic and late apoptotic cells than CuCl_2_ at the same Cu concentrations (*p* < 0.001, Figure S20). The enhanced apoptotic efficacy induced by FA-Au/Cu(II) PENPs should be ascribed to the combined effects of FA-mediated targeting specificity and Cu(II)-mediated CDT.

Based on the Cu(II)-mediated tumor CDT mechanism, which is capable of generating ROS via a Fenton-like reaction, the intracellular ROS levels triggered by FA-Au/Cu(II) PENPs in 4T1 cells were further quantified by DCFH-DA staining. As shown in Figure 4d, 4T1 cells with treatment of CuCl_2_ and FA-Au/Cu(II) PENPs for 5 h show higher levels of ROS compared to those treated with PBS, as evidenced by the enhanced fluorescence intensity. Furthermore, CuCl_2_ and FA-Au/Cu(II) PENPs could induce many more ROS in 4T1 cells at the higher Cu concentration. The statistical analysis of the intracellular relative ROS levels further confirms that FA-Au/Cu(II) PENPs could cause significantly higher ROS levels in 4T1 cells than CuCl_2_ at the same Cu concentrations (*p* < 0.001, Figure 4e). This suggests the superior efficiency of the Fenton-like reaction catalyzed by FA-Au/Cu(II) PENPs. Consistent with the results of the apoptosis assay, the intracellular ROS level result verifies that FA-Au/Cu(II) PENPs serve as a more potent ROS generator than CuCl_2_, ultimately triggering subsequent cell apoptosis.

Given the upregulation of ROS results in the consumption of GSH, it is crucial to assess the GSH depletion ability of FA-Au/Cu(II) PENPs by determining the GSH level in 4T1 cells post-treatment. Utilizing a GSH/GSSG Assay Kit for quantification, a decreasing trend in intracellular GSH levels of 4T1 cells can be observed as Cu concentration increases for both FA-Au/Cu(II) PENPs- and CuCl_2_-treated cells. Notably, 4T1 cells treated with FA-Au/Cu(II) PENPs exhibit lower intracellular GSH levels than those treated with CuCl_2_ at the same Cu concentrations, indicating the superior GSH depletion ability of FA-Au/Cu(II) PENPs (Figure 4f).

The upregulation of ROS and downregulation of GSH suggest that FA-Au/Cu(II) PENPs have the potential to induce intracellular LPO in 4T1 cells. Subsequently, we utilized C11-BODIPY^581/591^ as an oxidative-sensitive fluorescent probe to quantify the intracellular LPO levels in 4T1 cells post-treatment. Flow cytometric analysis reveals that cells treated with CuCl_2_ and FA-Au/Cu(II) PENPs exhibit higher fluorescence intensities than those treated with PBS, indicating the relatively high levels of LPO caused by CuCl_2_ and FA-Au/Cu(II) PENPs in 4T1 cells. Furthermore, the inductions of intracellular LPO by CuCl_2_ and FA-Au/Cu(II) PENPs are Cu-concentration-dependent (Figure 4g). The quantitative analysis shows that FA-Au/Cu(II) PENPs could cause significantly higher LPO levels in 4T1 cells than CuCl_2_ at the same Cu concentrations (*p* < 0.001, Figure 4h). Overall, the developed FA-Au/Cu(II) PENPs can effectively produce ROS, deplete GSH, and promote LPO. This cascade subsequently disrupts the redox homeostasis and ultimately induces cancer cell apoptosis in vitro.

### 3.7. Targeted CT/MR Imaging In Vivo and Biodistribution

**Figure 5:**
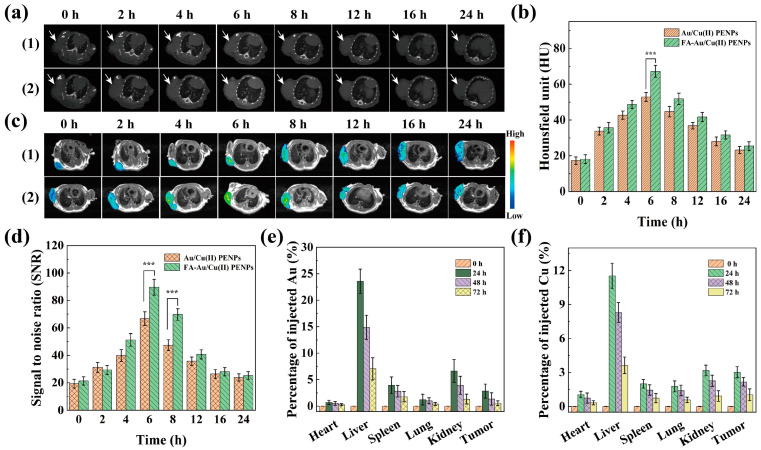
(**a**) Transverse CT images and (**b**) the CT values; (**c**) *T*_1_-weighted MR images and (**d**) the MR SNR values of xenografted 4T1 tumors at different time points after intravenous injection of (1) Au/Cu(II) PENPs and (2) FA-Au/Cu(II) PENPs, respectively. The white arrow in the figure points to the tumor site. The ICP-OES results of (**e**) Au and (**f**) Cu contents in the main organs and tumors of the 4T1-tumor-bearing mice at different time points after intravenous injection of FA-Au/Cu(II) PENPs (*** *p* < 0.001).

The remarkable CT/MR imaging performance of FA-Au/Cu(II) PENPs in vitro motivates us to explore their targeted CT/MR imaging potential of tumors in vivo using a 4T1 subcutaneously xenografted tumor model. Following intravenous administration of Au/Cu(II) PENPs and FA-Au/Cu(II) PENPs, respectively, the progressive enhancement (2–6 h post-injection) and gradual decrease (6–24 h post-injection) in CT imaging brightness of tumor (as indicated by the white arrow) can be observed in the 4T1-tumor-bearing mice (Figure 5a). However, these changes in CT imaging brightness of tumors are not obvious. Subsequently, the quantitative analysis of tumor CT values was further conducted, which revealed the identical results with the observed CT imaging brightness changes in tumor for both Au/Cu(II) PENPs- and FA-Au/Cu(II) PENPs-treated mice. Notably, FA-Au/Cu(II) PENPs-treated mice showed significantly higher tumor CT values than those of the Au/Cu(II) PENPs-treated mice at 6 h post-injection (*p* < 0.001), indicating the targeted accumulation of FA-Au/Cu(II) PENPs in tumors via FA-mediated binding specificity (Figure 5b). In terms of MR imaging of tumors in vivo, MR imaging of tumors reveals a similar change trend to the CT imaging of tumors over time, which attains the peak of MR signal intensity at 6 h post-injection for both the Au/Cu(II) PENPs- and FA-Au/Cu(II) PENPs-treated mice. From the *T*_1_-weighted MR pseudo-color images of the tumors, we can easily see the distinct differences in MR signal intensities at different time points based on the high sensitivity of MR imaging (Figure 5c). As shown in Figure 5d, the MR SNR values for tumors of the FA-Au/Cu(II) PENPs-treated mice are elevated to 89.7 and 69.8 at 6 h and 8 h post-injection, respectively, which are significantly higher than those for tumors of the Au/Cu(II) PENPs-treated mice at the corresponding time points (*p* < 0.001). This noticeable enhancement in tumor MR imaging signal illustrates the satisfactory tumor targeting ability of FA-Au/Cu(II) PENPs. Taken together, the above results demonstrate that FA-Au/Cu(II) PENPs could afford targeted CT/MR imaging of tumors in vivo.

Subsequently, to figure out the biodistribution of FA-Au/Cu(II) PENPs in vivo, the Au and Cu contents in major organs and tumors at different time intervals post intravenous injection were analyzed using ICP-OES. As illustrated in Figure 5e,f, Au and Cu elements primarily accumulate in the liver and kidney of mice, reaching their peaks of accumulation at 24 h post-injection. Subsequently, the Au and Cu contents in all tissues gradually decrease within 24–72 h post-injection. Until 72 h, their contents in all tissues nearly revert to pre-injection levels, indicating the favorable metabolic clearance effect of FA-Au/Cu(II) PENPs.

### 3.8. CDT of Tumor In Vivo and Biosafety Evaluation

**Figure 6:**
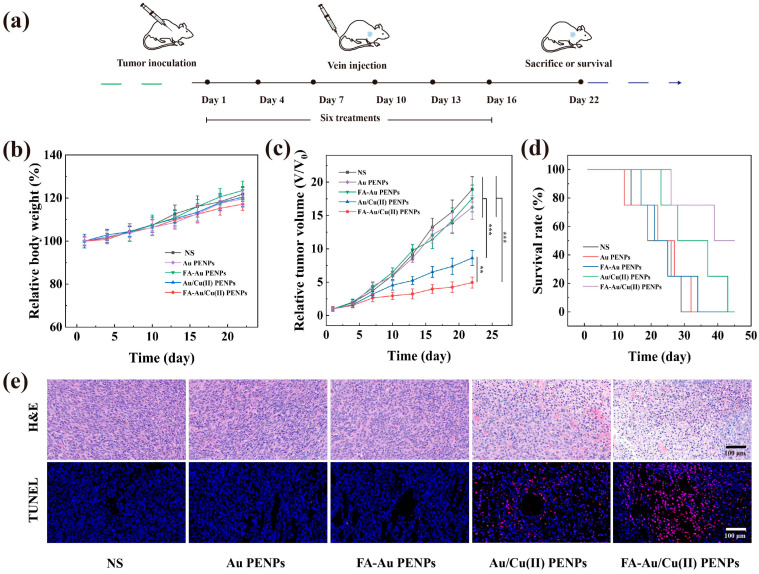
(**a**) Schematic illustration of FA-Au/Cu(II) PENPs-mediated CDT in vivo. (**b**) The relative body weight, (**c**) average tumor volume growth, and (**d**) survival rate of 4T1-tumor-bearing mice after different treatments. Typical photographs of the (**e**) H&E and TUNEL staining of xenografted 4T1 tumor tissues following various treatments. The scale bar represents 100 μm (** *p* < 0.01 and *** *p* < 0.001).

Finally, to assess the inhibition effect of Cu(II)-based nanocomposite-medicated CDT on 4T1 tumors, 4T1 subcutaneously xenografted tumor models were constructed and administered with FA-Au/Cu(II) PENPs (Figure 6a). After being administered with NS, FA-Au PENPs, Au PENPs, FA-Au/Cu(II) PENPs, and Au/Cu(II) PENPs, respectively, the relative body weights of 4T1-tumor-bearing mice in each group increase slightly, indicating that these nanocomposites have no potential toxicity that affects the growth of mice (Figure 6b). An analysis of tumor growth reveals that 4T1-tumor-bearing mice administered with FA-Au PENPs and Au PENPs demonstrate a tendency for the relative tumor volume to rapidly increase, similar to that of NS-treated mice. In contrast, the relative tumor volumes of mice administered with FA-Au/Cu(II) PENPs and Au/Cu(II) PENPs demonstrate a tendency to slowly increase, indicating Cu(II)-based nanocomposite-medicated tumor suppression efficacy based on the CDT mechanism (Figure S21). After 22 days of administration, both the FA-Au/Cu(II) PENPs and Au/Cu(II) PENPs display significantly higher tumor suppression efficiency than NS, FA-Au PENPs, and Au PENPs (*p* < 0.001). Furthermore, the relative tumor volume of mice administered with FA-Au/Cu(II) PENPs (4.95 ± 0.82 times) is significantly smaller than that of mice administered with Au/Cu(II) PENPs (8.63 ± 1.12 times, *p* < 0.01) after 22 days of administration, indicating the FA-mediated superior anti-cancer efficacy of FA-Au/Cu(II) PENPs in vivo (Figure 6c). The best tumor suppression efficiency of FA-Au/Cu(II) PENPs can be further verified by the survival rates of the 4T1-tumor-bearing mice. As described in Figure 6d, mice administered with FA-Au/Cu(II) PENPs demonstrate the highest survival rate (50%) among all of the mice, with treatments on the 45th day after administration.

The cell necrosis levels of ex vivo tumors after treatments were ultimately analyzed using H&E and TUNEL staining. As revealed in Figure 6e, pathological sections of tumor after treatment with FA-Au/Cu(II) PENPs display the largest area of cell necrosis and apoptosis among all the pathological sections with treatments, in both H&E and TUNEL staining images. Furthermore, the quantitative results of tumor apoptosis rate recorded from TUNEL staining images reveal that FA-Au/Cu(II) PENPs could induce a much higher apoptosis rate (57.92%) than that of Au/Cu(II) PENPs (21.63%, *p* < 0.001), FA-Au PENPs (1.21%, *p* < 0.001), Au PENPs (0.38%, *p* < 0.001), and NS (0.44%, *p* < 0.001), respectively (Figure S22). The results further suggest the best tumor suppression efficiency of FA-Au/Cu(II) PENPs, in keeping with the tumor growth results shown in Figure 6c.

**Figure 7:**
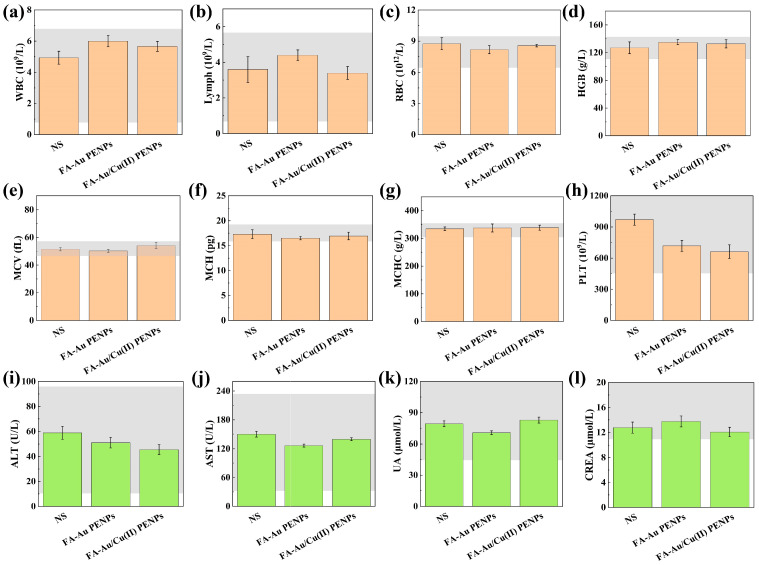
The blood routine analysis including levels of (**a**) white blood cells (WBCs), (**b**) lymphocytes (Lymph), (**c**) red blood cells (RBCs), (**d**) hemoglobin (HGB), (**e**) mean corpuscular volume (MCV), (**f**) mean corpuscular hemoglobin (MCH), (**g**) mean corpuscular hemoglobin concentration (MCHC), and (**h**) platelets (PLTs); the blood biochemistry analysis including (**i**) alanine aminotransferase (ALT), (**j**) aspartate aminotransferase (AST), (**k**) uric acid (UA), and (**l**) creatinine (CREA) of healthy nude mice after being injected with NS, FA-Au PENPs, and FA-Au/Cu(II) PENPs for 7 days. The gray-shaded region within the figure delineates the normal value range of the corresponding parameter.

The biosafety evaluation of the developed nanocomposites was subsequently conducted by H&E staining, blood routine, and serum biochemistry analysis. As revealed in Figure S23, no appreciable damage can be found in the main organ slices after administration with different nanocomposites through comparison with those administered with NS, suggesting that the developed nanocomposites do not cause organ toxicity. Furthermore, blood routine and serum biochemistry indexes of healthy nude mice after administration with FA-Au PENPs and FA-Au/Cu(II) PENPs, respectively, are all within the normal reference ranges, thus indicating the satisfactory biocompatibility of both FA-Au PENPs and FA-Au/Cu(II) PENPs (Figure 7).

## 4. Conclusions

Overall, the Cu(II)-based nanocomposites FA-Au/Cu(II) PENPs consisting of Cu(II) and Au NPs were synthesized with a facile method for targeted CT/MR imaging-guided CDT of tumors in this study. The multifunctional PEI.NH_2_-entrapped Au NPs could complex Cu(II) to form nanocomposites, representing an attractive design of incorporating simple modules into these all-in-one theranostic nanoformulations. The prepared FA-Au/Cu(II) PENPs possess favorable stability of colloidal dispersion, pH-sensitive sustainable Cu(II) release ability, and Fenton-like catalytic activity specifically under an excessive GSH and endogenous H_2_O_2_ environment. Furthermore, the emerging FA-Au/Cu(II) PENPs exhibit the capability to be specifically delivered to FAR-overexpressing cancer cells, where they effectively produce ROS, deplete GSH, promote LPO, and ultimately induce cancer cell apoptosis in vitro. Above all, the Cu(II)-based nanocomposites FA-Au/Cu(II) PENPs could afford complementary targeted CT/MR dual-mode imaging and CDT of cancer in vitro and in vivo, processing safe metabolism without distinct systemic toxicity. This Cu(II)-based nanocomposite paradigm inspires the construction of advanced theranostic nanoplatforms incorporating alternative transition metal ions.

## Data Availability

Data are contained within the article and Supplementary Materials.

## Supplementary Materials

The following supporting information can be downloaded at https://www.mdpi.com/article/10.3390/polym17030423/s1: Table S1: Hydrodynamic sizes, polydispersity indexes (PDIs), and zeta potentials of FA-Au PENPs and FA-Au/Cu(II) PENPs; Table S2: Zeta potentials of FA-Au/Cu(II) PENPs dissolved in H_2_O and PBS at different time points, respectively; Table S3: The IC_50_ values of FA-Au/Cu(II) PENPs, Au/Cu(II) PENPs, and CuCl_2_ towards 4T1 cells after 24 h treatment; Figure S1: ^1^H NMR spectra of (a) FA-PEG-COOH, (b) PEI.NH_2_-PA, (c) PEI.NH_2_-(PEG-FA)-PA, (d) PEI.NH_2_-FI-(PEG-FA)-PA, (e) PEI.NH_2_-*m*PEG-PA, and (f) PEI.NH_2_-FI-*m*PEG-PA dissolved in D_2_O, respectively; Figure S2: UV-vis spectra of FA-PEG-COOH, PEI.NH_2_-PA, PEI.NH_2_-(PEG-FA)-PA, and PEI.NH_2_-FI-(PEG-FA)-PA dissolved in water, respectively; Figure S3: UV-vis spectra of (a) PEI.NHAc-FI-(PEG-FA)-PA and (c) PEI.NHAc-FI-*m*PEG-PA complexed with different molar equivalents of Cu(II), respectively. The fitted curve of the absorbance at a certain wavelength versus the molar ratio (b) between Cu(II) and PEI.NHAc-FI-(PEG-FA)-PA and the molar ratio (d) between Cu(II) and PEI.NHAc-FI-*m*PEG-PA; Figure S4: UV-vis spectra of CuCl_2_, PEI.NHAc-FI-(PEG-FA)-PA, and PEI.NHAc-FI-(PEG-FA)-PA/60Cu(II) dissolved in water; Figure S5: UV-vis spectrum and photograph of FA-Au/Cu(II) PENPs dissolved in water; Figure S6: (a) Histogram of particle size distribution, (b) high-resolution TEM image, and (c) selected-area electron diffraction (SAED) pattern of FA-Au/Cu(II) PENPs; Figure S7: XPS spectrum of Cu 2p region of FA-Au/Cu(II) PENPs; Figure S8: FT-IR spectra of FA-Au PENPs and FA-Au/Cu(II) PENPs; Figure S9: Hydrodynamic size distributions of (a) FA-Au PENPs and (b) FA-Au/Cu(II) PENPs; Figure S10: UV-vis spectra of FA-Au/Cu(II) PENPs (a) at different time points and (b) under different temperature conditions, respectively; Figure S11: (a) Hydrodynamic sizes and (b) PDI of FA-Au/Cu(II) PENPs dissolved in H_2_O and PBS at different time points, respectively; Figure S12: Hemolysis rate and the photograph of red blood cells after being treated with different Cu concentrations of FA-Au/Cu(II) PENPs for 2 h. The red blood cells treated with H_2_O and PBS were used as positive and negative controls, respectively. The centrifuge tube containing FA-Au/Cu(II) PENPs ([Cu] = 40 μg/mL) solution without red blood cells is shown on the rightmost side of the photograph; Figure S13: Flow cytometric analysis of (a) 4T1-HFAR cells treated with PBS and (b) 4T1-LFAR, (c) 4T1-HFAR cells treated with Au/Cu(II) PENPs and (d) 4T1-LFAR, and (e) 4T1-HFAR cells treated with FA-Au/Cu(II) PENPs for 2 h, respectively; Figure S14: Flow cytometric determination of (a) the mean fluorescence and (b) the percentage of 4T1 cells taken up with the nanocomposites after different treatments for 2 h, respectively; Figure S15: ICP-OES result of Au uptake by 4T1-LFAR and 4T1-HFAR cells treated with FA-Au/Cu(II) PENPs at different Au concentrations (0, 50, 100, 200 μM) for 2 h, respectively; Figure S16: (a) CT images of (1) FA-Au/Cu(II) PENPs and (2) Omnipaque at different Au or I concentrations; (b) *T*_1_-weighted MR pseudo-color images of (1) FA-Au/Cu(II) PENPs and (3) CuCl_2_ at different Cu concentrations; Figure S17: Inverted microscopic images of 4T1 cells after 24 h incubation with (a) PBS, (b) Au PENPs, and (c) FA-Au PENPs at PEI.NH_2_ concentration of 16 μM, (d) CuCl_2_, (e) Au/Cu(II) PENPs, and (f) FA-Au/Cu(II) PENPs at Cu concentration of 1000 μM, respectively; Figure S18: Cell viability of L929 cells after 24 h incubation with (a) FA-Au PENPs and Au PENPs at different PEI.NH_2_ concentrations, as well as (b) FA-Au/Cu(II) PENPs, Au/Cu(II) PENPs, and CuCl_2_ at different Cu concentrations; Figure S19: Cell viability of 4T1-LFAR and 4T1-HFAR cells with 2 h incubation of FA-Au/Cu(II) PENPs and Au/Cu(II) PENPs at Cu concentration of 1000 μM, followed by 24 h incubation in fresh medium. PBS-incubated 4T1-HFAR cells were set as the control; Figure S20: The flow cytometric assay performed using Annexin V-FITC/PI double staining of 4T1 cells after 24 h incubation with different Cu concentrations of CuCl_2_ and FA-Au/Cu(II) PENPs. The percentages of (a) intact cells, (b) early apoptotic cells, and (c) late apoptotic cells were recorded, respectively; Figure S21: Individual tumor growth curves of 4T1-tumor-bearing mice treated with (a) NS, (b) Au PENPs, (c) FA-Au PENPs, (d) Au/Cu(II) PENPs, and (e) FA-Au/Cu(II) PENPs, respectively; Figure S22: The apoptosis rate of tumor tissues with different treatments recorded from TUNEL stained sections; Figure S23: H&E staining of the major organs of 4T1-tumor-bearing mice after treatment with different nanoparticles, respectively.
