# Supplementary material for: Copper(II)-Complexed Polyethylenimine-Entrapped Gold Nanoparticles Enable Targeted CT/MR Imaging and Chemodynamic Therapy of Tumors
Source: Polymers (Basel). 2025 Feb 6;17(3):423. doi: 10.3390/polym17030423 (PMC11819761; doi:10.3390/polym17030423)
Supplement: Supplementary file 1 [file polymers-17-00423-s001.zip › polymers-3439719-supplementary.pdf]

Supporting Information

Copper(II)-Complexed Polyethylenimine-Entrapped Gold Nanoparticles Enable Targeted CT/MR Imaging and Chemodynamic Therapy of Tumors

Lingxiu He[1], *Na Liu*[1], *Risong Pan*[2], *and Jingyi Zhu*[1,*]

1 School of Pharmaceutical Sciences, Nanjing Tech University, Nanjing 211816, China

2 College of Biotechnology and Pharmaceutical Engineering, Nanjing Tech University, Nanjing 211816, China

* Correspondence: zhujy1210@njtech.edu.cn

2. Materials and Methods

2.1. Materials

Amine group and carboxyl group terminal polyethylene glycol (NH_2-PEG-COOH, Mw = 2000) and carboxyl group terminal PEG monomethyl ether (*m*PEG-COOH, Mw = 2000) were obtained from Shanghai Yayi Biotechnology Corporation (Shanghai, China). Folic acid (FA), 2-picolinic acid (PA-COOH), cupric chloride dihydrate ($CuCl_2 \cdot 2H_2O$), glutathione (GSH), chloroauric acid ($HAuCl_4$), and 2,9-dimethyl-1,10-phenanthroline hemihydrate (neocuproine) were purchased from Shanghai Macklin Biochemical Technology Co., Ltd. (Shanghai, China). Hyperbranched polyethylenimine (PEI.NH_2, Mw = 25000), 1-ethyl-3-(3-(dimethylamino)propyl) carbodiimide hydrochloride (EDC), and N-hydroxy succinimide (NHS) were provided from Sigma-Aldrich (St. Louis, MO). Dimethyl sulfoxide (DMSO), dimethylformamide (DMF), and 5,5'-dithiobis-(2-nitrobenzoic acid) (DTNB) were purchased from Shanghai Aladdin Biochemical Technology Co., Ltd. (Shanghai, China). Fluorescein isothiocyanate (FI), phosphate buffered saline (PBS), normal saline (NS), oxidized GSH (GSSG), and regenerated cellulose dialysis membranes with the molecular weight cut-off (MWCO) of 1000 and 8000-14000 were purchased from Shanghai Yuanye Biotechnology Co., Ltd. (Shanghai, China). Triethylamine and acetic anhydride were from Sinopharm Chemical Reagent Co., Ltd. (Shanghai, China). Sodium borohydride ($NaBH_4$) was purchased from Shanghai Ebi Chemical Reagent Co., Ltd (Shanghai, China). Hydrogen peroxide (H_2O_2) was purchased from Shanghai Lingfeng Chemical Reagent Co., Ltd (Shanghai, China). Methylene blue (MB) was from Shanghai Titan Technology Co., Ltd (Shanghai, China). Dulbecco's modified eagle medium (DMEM), minimum essential medium (MEM), and fetal bovine serum (FBS) were purchased from Gibco (Carlsbad, CA). Penicillin and streptomycin were from Gino Biomedical Technology Co., Ltd. (Hangzhou, China). Cell Counting

Kit-8 (CCK-8) was purchased from 7Sea Biotech Co., Ltd. (Shanghai, China). Annexin V-labeled fluorescein isothiocyanate (V-FITC)/propidium iodide (PI) Apoptosis Kit, 2′,7′-dichlorofluorescin diacetate (DCFH-DA), and GSH/GSSG Assay Kit were provided by Beyotime Biotechnology (Shanghai, China). Oxidative-sensitive fluorescent probe C11-BODIPY$^{581/591}$ was from GlpBio Technology (Montclair, CA). Water used in all experiments was purified using a Milli-Q Plus 185 water purification system (Millipore, Bedford, MA) with a resistivity higher than 18.2 MΩ·cm.

2.2. Synthesis of FA-PEG-COOH

The functional molecule FA-PEG-COOH was synthesized through the amidation reaction between NH_2-PEG-COOH and FA referring to a specific procedure described in the previous literature [1]. Briefly, FA (26.48 mg, 60.00 μmol) dispersed in DMSO (7 mL) was first activated by the DMSO solution containing equal molar equivalent of EDC (11.50 mg, 60.00 μmol, 5 mL) and NHS (6.91 mg, 60.00 μmol, 5 mL). After 3 h magnetic stirring, the activated FA was coupled with the amine group of NH_2-PEG-COOH (80.00 mg, 40.00 μmol) dissolved in 20 mL DMSO at the 1.5:1 molar ratio between FA and NH_2-PEG-COOH under stirring condition for 72 h. Ultimately, the above reaction mixture was dialyzed in water (9 times, 2 L) through a dialysis membrane with MWCO of 1000 for 3 days to remove the excess reactants or by-products, followed by lyophilization to obtain FA-PEG-COOH (yield = 84.2%).

2.3. Synthesis of PEI.NH$_2$-FI-(PEG-FA)-PA

Subsequently, the multifunctional polyethylenimine was synthesized following the preparation procedure depicted in Figure 1a. Briefly, PA-COOH (19.70 mg, 0.160 mmol) dissolved in DMF (10 mL) was first activated by the DMF solution containing EDC (306.72 mg, 1.60 mmol, 25 mL, 10 molar equivalents) and NHS (184.16 mg, 1.60 mmol, 10 mL, 10 molar equivalents) under intensive

magnetic stirring for 3 h. The above prepared PA-COOH/EDC/NHS mixture was then added dropwise into the 15 mL aqueous solution of PEI.NH_2 (50.00 mg, 2.00 μmol) following the 80:1 molar ratio between PA-COOH and PEI.NH_2, and the mixture was continuously stirred for 72 h at room temperature, yielding PEI.NH_2-PA (yield = 79.5%). Subsequently, using the same EDC/NHS coupling chemistry as above, the prepared 8 mL aqueous solution of FA-PEG-COOH (47.59 mg, 21.52 μmol, 20 molar equivalents) was activated and then reacted with PEI.NH_2-PA (29.50 mg, 1.076 μmol, 20 mL) solution for 72 h to generate PEI.NH_2-(PEG-FA)-PA (yield = 86.2%). Finally, 2 mL DMSO containing FI (1.60 mg, 4.11 μmol, 5 molar equivalents) was reacted with the 15 mL DMSO solution of PEI.NH_2-(PEG-FA)-PA (50.70 mg, 0.82 μmol) for 24 h in a light-avoiding environment to finish the preparation of PEI.NH_2-FI-(PEG-FA)-PA (yield = 82.7%). Concurrently, FA-free nanoparticles PEI.NH_2-FI-*m*PEG-PA (yield = 78.9%) were synthesized under the similar conditions, except that the *m*PEG-COOH was employed in place of FA-PEG-COOH.

2.4. Formation of FA-Au/Cu(II) PENPs

The synthesized PEI.NH_2-FI-(PEG-FA)-PA was subsequently employed to entrap Au NPs, acetylate the residual amine groups of PEI.NH_2, and complex with Cu(II), referring to the methods published with slight modifications [2-4]. Briefly, 100 molar equivalents of $HAuCl_4$ solution (0.626 mL, 30 mg/mL, 45.60 μmol) were slowly added into PEI.NH_2-FI-(PEG-FA)-PA aqueous solution (28.50 mg, 0.456 μmol, 15 mL) and then stirred for 0.5 h. Subsequently, $NaBH_4$ aqueous solution (5.18 mg, 136.80 μmol, 1 mL) was rapidly added into the above reaction mixture at a 3:1 molar ratio between $NaBH_4$ and $HAuCl_4$, and the solution was stirred continuously for 2 h to generate the [$(Au^0)_{100}$-PEI.NH_2-FI-(PEG-FA)-PA] NPs. Thereafter, triethylamine (30.78 μL, 218.89 μmol) and acetic anhydride (20.90 μL, 218.89 μmol) with 3 times molar excess of the surface amines of PEI.NH_2

were utilized to attain acetylation *via* an additional 24 h amidation reaction. The acquired [$(Au^0)_{100}$-PEI.NHAc-FI-(PEG-FA)-PA] NPs (denoted as, FA-Au PENPs) aqueous solution was subject to dialysis using a dialysis membrane with MWCO of 8000-14000 for 3 days to remove the excess reactants or by-products, followed by lyophilization to obtain FA-Au PENPs powder (yield = 88.5%). Finally, FA-Au PENPs (15.00 mg, 0.175 μmol) dispersed in water (20 mL) were mixed with 1 mL $CuCl_2$ aqueous solution (1.80 mg, 10.50 μmol) at an optimal Cu(II) concentration (60 molar equivalents) under ultrasonic stirring for 10 min. After freeze-drying process, FA-Au/Cu(II) PENPs were obtained (yield = 91.9%). Concurrently, FA-free nanocomposites Au/Cu(II) PENPs (yield = 88.6%) were synthesized using PEI.NH_2-FI-*m*PEG-PA as a nanoplatform under similar conditions to facilitate further targeted comparative analysis.

2.5. Characterization Techniques

Proton nuclear magnetic resonance (^{1}H NMR) spectra were recorded using a Bruker AV-400 NMR spectrometer. All samples were dissolved in D_2O before measurements. Ultraviolet-visible (UV-vis) spectra were collected using a Lambda 25 UV-vis spectrophotometer (Perkin Elmer, Waltham, MA, USA). Fourier transform infrared (FT-IR) spectra were recorded on a Nicolet 6700 FTIR spectrophotometer (Thermo Electron Corporation, Madison, WI, USA). Samples were mixed with milled KBr crystals and pressed to form 13-mm diameter disks before measurements. Transmission electron microscopy (TEM) imaging was executed using a JEOL 2010F analytical electron microscope (JEOL, Tokyo, Japan) operating at 200 kV. An aqueous solution of FA-Au/Cu(II) PENPs (6 μL, 1 mg/mL) was dropped onto a carbon-coated copper grid and air-dried before measurement. The size distribution histogram of the FA-Au/Cu(II) PENPs was measured using ImageJ software (https://imagej.nih.gov/ij/download.html). More than 300 NPs from TEM images were randomly

selected to analyze the size. Zeta potential and dynamic light scattering (DLS) measurements were tested using a Malvern Zetasizer Nano ZS90 system (Worcestershire, UK) coupled with a standard laser with a wavelength of 633 nm. X-ray photoelectron spectroscopy (XPS) measurements were performed using PHI 5000 Versa Probe X-ray photoelectron spectrometer (a monochromatic Al K Alpha X-ray radiation).

2.6. Determination of Cu(II) Complexing Capacity

To determine the Cu(II) complexing capacity of multifunctional polyethylenimine derivatives, specifically PEI.NHAc-FI-(PEG-FA)-PA and PEI.NHAc-FI-*m*PEG-PA, the PEI.NHAc-FI-(PEG-FA)-PA/Cu(II) complexes and PEI.NHAc-FI-*m*PEG-PA/Cu(II) complexes with a series of Cu(II) concentrations were synthesized respectively. Briefly, different amounts of $CuCl_2$ aqueous solution (20-180 molar equivalents of $CuCl_2$ per PEI.NH_2) were respectively added into PEI.NHAc-FI-(PEG-FA)-PA or PEI.NHAc-FI-*m*PEG-PA solution under ultrasonic conditions, and the mixtures were allowed to react for 10 min. Through observing the changes of characteristic absorption peak and quantitatively analyzing the relationship between absorbance at the characteristic peak wavelength and molar equivalents of Cu(II) by UV-vis spectroscopy (Perkin Elmer, Waltham, MA, USA), the optimal complexing equivalent of Cu(II) in PEI.NHAc-FI-(PEG-FA)-PA/Cu(II) complexes and PEI.NHAc-FI-*m*PEG-PA/Cu(II) complexes can be determined.

2.7. In Vitro Stability Study

The colloidal stability of the formed FA-Au/Cu(II) PENPs was investigated by UV-vis spectroscopy and DLS measurements according to the methods published in our previous articles [2,5]. UV-vis spectroscopy was utilized to evaluate the surface plasmon resonance (SPR) peak changes of the FA-Au/Cu(II) PENPs dispersion which set in various temperature conditions (4 °C, 25 °C, 37 °C,

and 50 °C) and time points (day 1, day 3, day 5, and day 7) to reflect their colloidal stability. In addition, the hydrodynamic sizes, polydispersity indexes (PDIs), and zeta potentials of FA-Au/Cu(II) PENPs dispersed in water and PBS at different time points (day 1, day 3, day 5, and day 7) were also tested *via* DLS measurements. Through monitoring the data change trends within 7 days, the colloidal stability was determined.

2.8. Hemolysis Assay

Hemolysis assay was performed to evaluate the hemocompatibility of the FA-Au/Cu(II) PENPs according to the literature [6]. All animal experiments were performed in accordance with the guidelines for Care and Use of Laboratory Animals of Nanjing Tech University and approved by the Animal Ethics Committee of Nanjing Tech University (Ethical certificate number: IACUC-20240310-12). Firstly, whole blood (2 mL) obtained *via* orbital blood extraction from healthy nude mice was added into a centrifuge tube containing heparin sodium, followed by centrifuging (3000 rpm, 5 min), washing and discarding the supernatant to collect red blood cells. The prepared red blood cell suspension (100 μL) diluted with PBS for 10 times was respectively added into centrifuge tubes containing 900 μL of ultrapure water (positive control), PBS (negative control) and PBS solution of FA-Au/Cu(II) PENPs with a series of Cu(II) concentrations (2.5, 5, 10, 20, and 40 μg/mL). After 2 h incubation at 37 °C, the mixtures were centrifuged at 1.0×10^4 rpm for 5 min. The photograph of samples was taken and the absorbance of supernatant in every sample at 540 nm was tested by UV-vis spectroscopy (Perkin Elmer, Waltham, MA, USA). The hemolysis rate was finally calculated referring to the formula published in the article [6].

2.9. Cu(II) Release Property in Vitro

Triplicate samples of FA-Au/Cu(II) PENPs (1 mg) were individually dispersed in 1 mL of

phosphate-citrate buffer with different pH values (5.0, 6.5, and 7.4). Each FA-Au/Cu(II) PENPs dispersion was then sealed in dialysis bags (MWCO = 8000-14000) and placed in 20 mL of the corresponding phosphate-citrate buffer with the specific pH. The release devices were subsequently put in a vapor-bathing vibrator that maintained a constant temperature of 37 °C. At designated time intervals, 1 mL of the sample from the outer phase buffer medium was collected and supplemented with an equivalent volume of the phosphate-citrate buffer with the corresponding pH. Following this, the Cu(II) concentration in the collected samples was measured by Leeman Prodigy inductively coupled plasma-optical emission spectroscopy (ICP-OES, Hudson, NH, USA) after digestion with aqua regia.

2.10. Reduction of Cu(II) to Cu(I)

To determine the process wherein Cu(II) in FA-Au/Cu(II) PENPs could react with GSH to generate GSSG and Cu(I), FT-IR and UV-vis spectroscopies were utilized to verify the initial step of the chemodynamic therapy (CDT) mechanism. Firstly, the GSH solution (2 mL, 10 mM) was mixed with the FA-Au/Cu(II) PENPs solution (2 mL, [Cu] = 10 mM). After a 10 min reaction, the supernatant of the reaction mixture was collected, freeze-dried, and characterized using FT-IR spectroscopy. Purchased GSH and GSSG were used as controls for analysis and comparison. The method is consistent with the previous protocols published in the literature [3].

To further confirm the generation of Cu(I) resulting from the reduction of Cu(II), neocuproine was employed as an indicator due to its exhibition of a characteristic absorption peak in UV-vis spectroscopy upon reacting with Cu(I). Briefly, the FA-Au/Cu(II) PENPs solution (100 μL, [Cu] = 1 mM) mixed with or without GSH solution (100 μL, 1 mM). After a 5 min reaction, neocuproine (200 μL, 0.2 mg/mL) dispersed in methanol was added and incubated for 5 min. Neocuproine with the same

concentration was used as control for comparison. Finally, all the samples were detected by UV-vis spectroscopy (Perkin Elmer, Waltham, MA, USA).

2.11. FA-Au/Cu(II) PENPs-Mediated GSH Depletion

The consumption of GSH induced by the released Cu(II) from FA-Au/Cu(II) PENPs was detected using DTNB as an indicator under different pH conditions according to the reported literature [7,8]. In detail, triplicate FA-Au/Cu(II) PENPs (100 μL, 50 μg/mL) were individually dispersed in PBS solution with different pH values (5.0, 6.5, and 7.4). Each of these solutions was then added to 20 μL of PBS solution containing GSH (0.6 mM) with the corresponding pH and allowed to react for 0.5 h. Subsequently, sodium hydroxide (200 μL, 0.15 mol/L) and formaldehyde (80 μL, 3%) solutions were added to the mixture and incubated for 2 min. DTNB (10 μL, 0.3 mg/mL) dissolved in methanol was added to the above mixture and incubated for an additional 3 min. Finally, the FA-Au/Cu(II) PENPs-mediated GSH depletion was determined by analyzing the mixture's absorbance at 412 nm using UV-vis spectroscopy (Perkin Elmer, Waltham, MA, USA).

2.12. Generation of ·OH by Fenton-Like Reaction

The MB degradation measurements were performed referring to the previous literature [3]. Firstly, the requisite solutions such as FA-Au/Cu(II) PENPs ([Cu] = 10 mM), $CuCl_2$ (10 mM), GSH (10 mM), MB (10 μg/mL), and H_2O_2 (10 mM) were individually prepared. These solutions with specific concentrations were then mixed in equal volumes according to the following groups: FA-Au/Cu(II) PENPs + MB + GSH + H_2O_2, FA-Au/Cu(II) PENPs + MB, $CuCl_2$ + MB + GSH + H_2O_2, and $CuCl_2$ + MB. 2 h later, the MB degradation of the aforementioned solutions was analyzed by recording the absorbance at 665 nm using UV-vis spectroscopy (Perkin Elmer, Waltham, MA, USA). Meanwhile, the MB degradation levels of FA-Au/Cu(II) PENPs + MB + GSH + H_2O_2 at different time points (0,

30, 60, 90, and 120 min) were also recorded through UV-vis spectroscopy (Perkin Elmer, Waltham, MA, USA).

To assess the effect of pH environments on MB degradation of FA-Au/Cu(II) PENPs + MB + GSH + H_2O_2, we recorded the MB degradation over time under different pH conditions. Firstly, MB (10 μg/mL), H_2O_2 (10 mM), GSH (10 mM), and FA-Au/Cu(II) PENPs ([Cu] = 10 mM) were separately dissolved in PBS solution with different pH values (5.0, 6.5, and 7.4). Subsequently, equal amounts of FA-Au/Cu(II) PENPs, GSH, H_2O_2, and MB solution were thoroughly mixed at room temperature for each pH condition. After that, the absorbance of the mixtures at 665 nm was measured at various time points through UV-vis spectroscopy, and the MB residual percentage for each group was calculated statistically.

2.13. X-Ray Attenuation Measurements

X-ray attenuation characterization of FA-Au/Cu(II) PENPs was conducted using Omnipaque (an iodine-based contrast agent) as a reference. Firstly, aqueous solutions of FA-Au/Cu(II) PENPs and Omnipaque with a series of gold (Au) or iodine (I) concentration (0.01, 0.02, 0.04, 0.08 and 0.1 M) were prepared and then transferred into 0.5 mL centrifuge tubes, respectively. Subsequently, these samples were subjected to computed tomography (CT) imaging by a clinical Micro-CT imaging system (SkyScan 1176, Bruker, Berlin, Germany). The corresponding CT values were obtained to evaluate the X-ray attenuation performance of FA-Au/Cu(II) PENPs. The relevant parameters of the Micro-CT imaging system were set as 80 mA, 100 kV, and 0.625 mm slice thickness.

2.14. T_1 MR Relaxometry Measurements

T_1 MR relaxometry of FA-Au/Cu(II) PENPs was conducted by a 0.5 T NMI20-Analyst NMR analyzing and imaging system (Shanghai Niumag Corporation, Shanghai, China). The aqueous

solutions of $CuCl_2$ and FA-Au/Cu(II) PENPs with Cu(II) concentrations ranging from 0.05 mM to 0.8 mM were prepared. Subsequently, the as-prepared samples were subjected to MR scanning, followed by the measurement of T_1 relaxation times. The r_1 relaxivity was then calculated by linearly fitting the inverse T_1 relaxation time ($1/T_1$) as a function of Cu(II) concentration. The instrumental parameters were set referring to the literature [4].

2.15. Cell Culture

4T1 cells (a mouse breast cancer cell line) and L929 cells (a mouse fibroblast cell line) were obtained from the Institute of Biochemistry and Cell Biology, the Chinese Academy of Sciences (Shanghai, China). 4T1 cells were regularly cultured in DMEM medium supplemented with 10% FBS, penicillin (100 U/mL) and streptomycin (100 U/mL) in a cell incubator with 5% CO_2 at 37 °C. L929 cells were cultured under the similar conditions, except that MEM medium was employed in place of DMEM medium.

2.16. Targeted Cellular Uptake Assay

4T1 cells (2×10^5 cells/well) were seeded into 12-well plates and then divided into two groups. One group was treated with medium (1 mL) containing FA (2.5 μM), while the other group was treated with an equal volume of FA-free medium. After overnight incubation, 4T1 cells with low-level FA receptor expression (4T1-LFAR) and 4T1 cells with high-level FA receptor expression (4T1-HFAR) were generated, respectively. The procedure aligns with the previous protocols published in the literature [9].

The targeting specificity and targeted cellular uptake property of FA-Au/Cu(II) PENPs to 4T1-HFAR were then investigated *via* flow cytometry, confocal microscopy, and ICP-OES. Briefly, the adherent 4T1-HFAR or 4T1-LFAR cells were treated with medium containing PBS, Au/Cu(II) PENPs

or FA-Au/Cu(II) PENPs (5 μM) for 2 h. Following trypsinization and resuspension, the cells receiving different treatments were stored in PBS solution and analyzed using a FACScan analyzer (Becton Dickinson, Franklin, CA, USA) for fluorescence detection *via* flow cytometry. As for confocal microscopy, after 2 h treatment with different materials, the cells in each well were rinsed with PBS, fixed with 5% glutaraldehyde, counter stained with Hoechst 33342 (1 μg/mL), and imaged by confocal microscopy (Carl Zeiss LSM 700, Jena, Germany). In regards to ICP-OES test, the adherent 4T1-HFAR and 4T1-LFAR cells were treated with medium containing FA-Au/Cu(II) PENPs with different Au concentrations (0, 50, 100, and 200 μM) for 2 h, respectively. The targeted cellular uptake of Au in each cell sample was quantified by ICP-OES after digestion with aqua regia.

2.17. Targeted CT/MR Imaging in Vitro

4T1 cells (2×10^6 cells/well) were seeded into a 6-well plate and cultured overnight in a 5% CO_2 incubator maintained at a constant temperature of 37 °C. As for the CT imaging, the cells were treated with fresh medium containing different Au concentrations (0, 25, 50, 100, and 200 μM) of FA-Au/Cu(II) PENPs or Au/Cu(II) PENPs for 2 h. Later, the cells were rinsed, trypsinized, centrifuged, resuspended and transferred into 2 mL centrifuge tubes, and then scanned by a clinical Micro-CT imaging system (SkyScan 1176, Bruker, Berlin, Germany). An identical cell culture protocol was employed for MR imaging. After treatment with FA-Au/Cu(II) PENPs or Au/Cu(II) PENPs with various Cu(II) concentrations (0, 25, 50, 100, and 150 μM) for 2 h, the cells were finally collected and scanned by a 3.0-T MR system (Signa HDxt, GE Medical Systems, Milwaukee, WI, USA).

2.18. Cytotoxicity Assay and Cell Morphology Analysis

The CCK-8 assay was used to evaluate the inhibition efficiency of the Cu(II)-based nanocomposites towards 4T1 cells. First, 4T1 cells were seeded into a 96-well plate at a density of 1.0

$\times 10^4$ cells/well. After incubating overnight, the medium was discarded and replaced with 100 μL of fresh medium containing different concentrations of prepared materials (Au PENPs, FA-Au PENPs, $CuCl_2$, Au/Cu(II) PENPs, or FA-Au/Cu(II) PENPs), respectively. 24 h later, the medium in each well was replaced with serum-free medium (100 μL) containing CCK-8 solution (10 μL) and the cells were incubated for a further 4 h. The cell culture plate was then placed in a Multiskan MK3 ELISA reader (Thermo Scientific, Waltham, MA, USA), the absorbance at the detected wavelength (450 nm) was recorded, and the cell viability as well as the half-maximal inhibitory concentration (IC_{50}) were calculated. To further investigate the cytotoxicity of nanocomposites towards normal cell lines, CCK-8 assay was performed on normal mouse fibroblast L929 cells *via* the same method under similar conditions.

The morphological characteristics of 4T1 cells were investigated using an inverted microscope (DM IL LED, Leica, Wetzlar, Germany) after treatment with FA-Au/Cu(II) PENPs, Au/Cu(II) PENPs, and $CuCl_2$ at the same Cu(II) concentration ([Cu] = 1000 μM), as well as with FA-Au PENPs and Au PENPs at a comparable carrier concentration ([PEI.NH_2] = 16 μM), which corresponded to the carrier concentration of FA-Au/Cu(II) PENPs and Au/Cu(II) PENPs above.

2.19. Targeted Cancer Cell Inhibition in Vitro

To deeply study the competitive binding effect of FA to FA receptors on the surface of 4T1 cells, the targeted cancer cell inhibition of FA-Au/Cu(II) PENPs towards 4T1-LFAR and 4T1-HFAR cells was further studied using CCK-8 assay, referring to the method published in the previous literature [5]. 4T1-LFAR cells (1×10^4 cells/well) with FA pre-incubation and 4T1-HFAR cells (1×10^4 cells/well) without FA pre-incubation were generated and seeded into a 96-well plate according to the previous study [9]. After overnight incubation, the adherent 4T1-LFAR and 4T1-HFAR cells were

exposed to medium containing Au/Cu(II) PENPs or FA-Au/Cu(II) PENPs at the Cu concentration of 1000 μM for 2 h. Subsequently, the treated cells were rinsed with PBS and incubated with fresh medium for 24 h. PBS-incubated 4T1-HFAR cells were utilized as control. Finally, the cells with treatments were tested and analyzed according to the standard protocols of CCK-8 assay.

2.20. Cell Apoptosis Assay

The apoptosis level of 4T1 cells with treatment of FA-Au/Cu(II) PENPs was further studied through a cell sorting method by flow cytometry using Annexin V-FITC/PI Apoptosis Kit following the standard protocol of the manufacturer. To eliminate potential fluorescence interference from FI, FA-Au/Cu(II) PENPs without FI conjugation were prepared for cell apoptosis evaluation in this experiment. First, 4T1 cells (2×10^5 cells/well) were seeded in 12-well plates and cultured overnight. After the cell adherent growth, the medium was replaced with the medium containing FA-Au/Cu(II) PENPs or $CuCl_2$ ([Cu] = 10 or 100 μM) and incubated for 24 h. 4T1 cells with treatment of PBS were set as control. Following this, the cells in each well were trypsinized, washed, and collected. Subsequently, the cells in each well were resuspended in 195 μL of pre-cooled binding solution, then stained with Annexin V-FITC (5 μL) and PI (10 μL) in a light-avoiding environment for 15 min. Finally, 20,000 cells from each sample were counted and sorted by a FACScan analyzer (Becton Dickinson, Franklin, CA, USA) for cell apoptosis analysis.

2.21. Determination of Intracellular ROS and LPO Levels

To determine the intracellular reactive oxygen species (ROS) levels of 4T1 cells with treatment of FA-Au/Cu(II) PENPs, DCFH-DA was utilized as an oxidative-responsive fluorescent probe to test ROS generation. Consistent with the methodology used in the Annexin V-FITC/PI apoptosis assay, FA-Au/Cu(II) PENPs without FI conjugation were utilized here. Firstly, 4T1 cells (2×10^5 cells/well)

were seeded in a 6-well plate and cultured for adherent growth. Subsequently, each well of 4T1 cells was separately supplemented with medium containing FA-Au/Cu(II) PENPs or $CuCl_2$ with different Cu(II) concentrations (10 and 100 μM) and incubated for an additional 5 h. 4T1 cells with treatment of PBS were used as control. Following this, 4T1 cells were washed and then incubated with 1 mL of serum-free medium containing DCFH-DA (10 μM) for 40 min in a light-avoiding environment. Ultimately, the cell sample from each well was washed and detected by a FACScan analyzer (Becton Dickinson, Franklin, CA, USA). In terms of intracellular lipid peroxidation (LPO) analysis, the same cell culture procedures were performed, except that the oxidative-sensitive fluorescent probe C11-BODIPY$^{581/591}$ was utilized in place of DCFH-DA.

2.22. Determination of Intracellular GSH Level in Vitro

To investigate the GSH consumption ability of FA-Au/Cu(II) PENPs, the intracellular GSH level of 4T1 cells was detected by GSH/GSSG Assay Kit. Consistent with Annexin V-FITC/PI apoptosis assay, FA-Au/Cu(II) PENPs without FI conjugation were utilized in this test. Firstly, 4T1 cells were seeded into 6-well plates at the cell density of 2×10^6 cells/well and cultured overnight. Subsequently, the cells were exposed to fresh DMEM medium containing FA-Au/Cu(II) PENPs or $CuCl_2$ ([Cu] = 0, 10, 100 μM) for 5 h. Following washing, trypsinization, and resuspension, the cells were harvested to determine the intracellular GSH level using GSH/GSSG Assay Kit referring to the manufacturer's instruction.

2.23. Targeted CT/MR Imaging in Vivo and Biodistribution

Animal experiments were conducted in adherence to the standard protocols approved by the Animal Ethics Committee of Nanjing Tech University (Ethical certificate number: IACUC-20240310-12). Female nude mice (BALB/c-nu, 4-5 weeks, 18-22 g) were sourced from Shanghai Slac Laboratory

Animal Center (Shanghai, China). Xenograft models were initially established in BALB/c-nu mice by subcutaneously injecting approximately 5 × 10^6 4T1 cells/mouse into their right upper limbs. Upon reaching the tumor volume within the range of 0.5-1.0 cm^3, the mice were randomly allocated into two experimental groups for the subsequent *in vivo* CT/MR imaging studies, in accordance with the reported literature [2,4,10]. Before the imaging scan, each 4T1 tumor-bearing mouse was anesthetized and subsequently administered with 100 μL of normal saline (NS) containing FA-Au/Cu(II) PENPs or Au/Cu(II) PENPs *via* tail vein injection. For CT imaging, FA-Au/Cu(II) PENPs and Au/Cu(II) PENPs solutions with an Au concentration of 0.08 M were employed. As for MR imaging, FA-Au/Cu(II) PENPs and Au/Cu(II) PENPs solutions with a Cu(II) concentration of 8 mM were utilized. After the administration, CT and MR images of the 4T1 tumor-bearing mice were captured at various time points using a clinical Micro-CT imaging system (SkyScan 1176, Bruker, Berlin, Germany) and a 3.0-T MR system (Signa HDxt, GE Medical Systems, Milwaukee, WI, USA), respectively. The instrumental parameters were set referring to the literature [2,10]. The hounsfield units (HU) from CT imaging and the signal to noise ratios (SNR) from MR imaging of tumor sites at corresponding time points were recorded and analyzed ultimately.

With regard to biodistribution assessment *in vivo*, 100 μL of NS containing FA-Au/Cu(II) PENPs ([Cu] = 8 mM) was intravenously injected into each tumor-bearing mouse. At the predefined time points (0, 24, 48, and 72 h, respectively) post-injection, the mice were euthanized to harvest their main organs and tumors for ICP-OES analysis. The biodistribution of FA-Au/Cu(II) PENPs was determined by quantitative analysis of Au and Cu contents in these tissues according to the method reported in the literature [6].

2.24. CDT of Tumor in Vivo

Following the successful establishment of the xenograft models (designated as day 1), the mice were randomly assigned to five experimental groups (4 4T1 tumor-bearing mice/group), including NS, FA-Au PENPs, Au PENPs, FA-Au/Cu(II) PENPs, and Au/Cu(II) PENPs. Subsequently, 100 μL of NS, FA-Au/Cu(II) PENPs ([Cu] = 8 mM), Au/Cu(II) PENPs ([Cu] = 8 mM), FA-Au PENPs with the same carrier concentration of FA-Au/Cu(II) PENPs ([Cu] = 8 mM), and Au PENPs with the same carrier concentration of Au/Cu(II) PENPs ([Cu] = 8 mM) were intravenously administered to the mice of the corresponding groups, respectively. Treatment was initiated on day 1 and subsequently administered every 3 days for a total of 6 doses. Throughout the experimental period, body weight (W_t), survival rate, and tumor volume (V_t) of each 4T1 tumor-bearing mouse in every mice group were recorded to evaluate the biosafety and CDT efficiency of the Cu(II)-based nanocomposites following the methods published in the literature [1,4]. Upon completion of the administration, tumors and major organs were dissected and collected from a euthanized mouse in each mice group. Through fixing, dehydration, embedding, slicing, and staining with hematoxylin-eosin (H&E), the acquired tissue sections of major organs and tumors were observed to analyze the levels of cell necrosis. In addition, a terminal deoxynucleotidyl transferase dUTP nick end labeling (TUNEL) assay was utilized to locate the apoptotic areas and determine the apoptosis rate of the *ex vivo* tumors. The acquisition and staining procedures of the pathological sections were similar to the standard protocols published in previous studies [2,4,10].

2.25. Blood Routine and Serum Biochemistry Assay

To confirm the biosafety of the generated nanosystem, 100 μL of NS, FA-Au/Cu(II) PENPs ([Cu] = 8 mM), and FA-Au PENPs with the same carrier concentration of FA-Au/Cu(II) PENPs ([Cu] = 8 mM) were intravenously administered to healthy nude mice, respectively. At 7 days post-injection, the

mice were sacrificed and the blood samples were collected. First, the blood cell counts were performed on an automated blood cell counter (BC-2800 Vet Analyzers, Mindray, Shenzhen, China) for blood routine analysis, including white blood cells (WBCs), lymphocyte (Lymph), red blood cells (RBCs), hemoglobin (HGB), mean corpuscular volume (MCV), mean corpuscular hemoglobin (MCH), mean corpuscular hemoglobin concentration (MCHC), and platelets (PLTs). Then the blood samples were centrifugated at 3000-4000 rpm for 10 min to obtain the serum, and the serum biochemistry data including liver function markers (alanine aminotransferase (ALT) and aspartate aminotransferase (AST)), and kidney function markers (uric acid (UA) and creatinine (CREA)) were analyzed by Servicebio Technology Co., Ltd. (Wuhan, China).

2.26. Statistical Analysis

All data were presented as the mean ± standard deviation ($n \geq 3$). All experimental data were analyzed by a one-way ANOVA statistical method to determine the significance levels. Significance levels were indicated as follows: (*) for $p < 0.05$, (**) for $p < 0.01$, and (***) for $p < 0.001$, respectively.

Table S1. Hydrodynamic sizes, polydispersity indexes (PDI) and zeta potentials of FA-Au PENPs and FA-Au/Cu(II) PENPs. Data are provided as mean ± SD (n = 3).

Sample	Hydrodynamic size (nm)	PDI	Zeta potential (mV)
FA-Au PENPs	252.2 ± 8.5	0.34 ± 0.04	-14.2 ± 0.3
FA-Au/Cu(II) PENPs	222.2 ± 21.7	0.34 ± 0.02	2.5 ± 2.0

Table S2. Zeta potentials of FA-Au/Cu(II) PENPs dissolved in H_2O and PBS at different time points, respectively. Data are provided as mean ± SD (n = 3).

	Zeta potential（mV）			
Solvent	Day 1	Day 3	Day 5	Day 7
H_2O	2.53 ± 0.34	4.24 ± 0.41	5.01 ± 0.97	5.12 ± 0.40
PBS	-1.51 ± 0.57	-2.69 ± 0.09	-1.24 ± 0.34	-2.13 ± 0.22

Table S3. The IC_{50} values of FA-Au/Cu(II) PENPs, Au/Cu(II) PENPs and $CuCl_2$ towards 4T1 cells after 24 h treatment.

Sample	IC_{50} ([Cu]: μM)
FA-Au/Cu(II) PENPs	70.80
Au/Cu(II) PENPs	111.98
$CuCl_2$	82.91

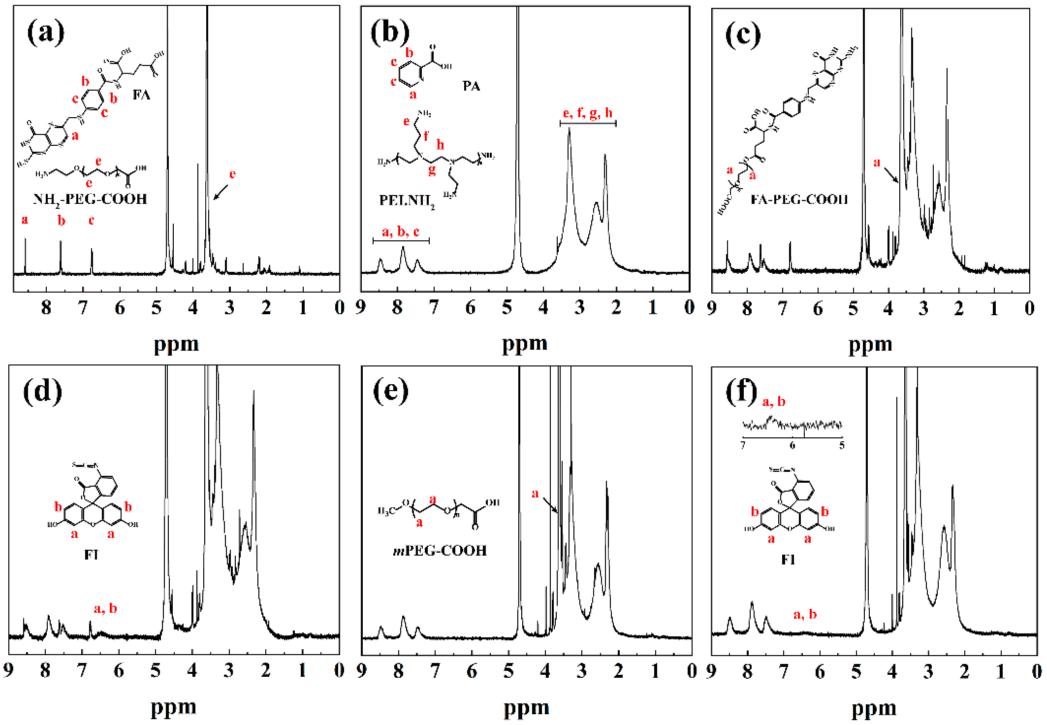

Figure S1. ^{1}H NMR spectra of (**a**) FA-PEG-COOH, (**b**) PEI.NH_2-PA, (**c**) PEI.NH_2-(PEG-FA)-PA, (**d**) PEI.NH_2-FI-(PEG-FA)-PA, (**e**) PEI.NH_2-*m*PEG-PA and (**f**) PEI.NH_2-FI-*m*PEG-PA dissolved in D_2O, respectively.

The peaks at 6.7 ppm, 7.6 ppm, and 8.6 ppm, which are associated with the characteristic peaks of FA, emerged in the ^{1}H NMR spectrum of FA-PEG-COOH, indicating the successful conjugation of FA to NH_2-PEG-COOH (Figure S1a). Following the linking of PA-COOH, FA-PEG-COOH, and FI onto PEI.NH_2, the characteristic peaks of PA-COOH (7.4 ppm, 7.8 ppm, and 8.4 ppm), FA-PEG-COOH (3.4-3.7 ppm), and FI (6.5 ppm) appeared in the ^{1}H NMR spectra of PEI.NH_2-PA, PEI.NH_2-(PEG-FA)-PA and PEI.NH_2-FI-(PEG-FA)-PA, respectively, indicating the successful conjugation of PA-COOH, FA-PEG-COOH, and FI moieties to PEI.NH_2. These results suggest the successful preparation of PEI.NH_2-PA, PEI.NH_2-(PEG-FA)-PA and PEI.NH_2-FI-(PEG-FA)-PA (Figure S1b, c, and d). Furthermore, through quantitative analysis of characteristic peaks in these ^{1}H NMR spectra, it can be determined that approximately 23.0 PA-COOH, 15.6 FA-PEG-COOH, 7.8 FA, and 2.2 FI

moieties were conjugated to each PEI.NH_2 using a method similar to that published in the literature [2-4]. Concurrently, FA-free nanoparticles PEI.NH_2-FI-*m*PEG-PA were also characterized using the same method. Through analyzing the ^{1}H NMR spectra of PEI.NH_2-*m*PEG-PA and PEI.NH_2-FI-*m*PEG-PA, it can be determined that the comparable functional moieties (15.8 *m*PEG-COOH and 2.0 FI) were present on PEI.NH_2-FI-*m*PEG-PA (Figure S1e, f).

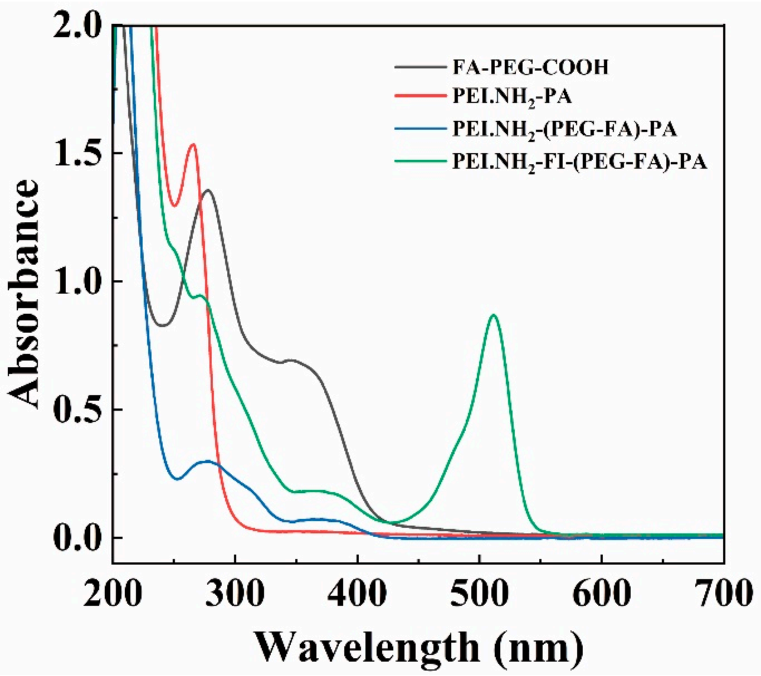

Figure S2. UV-vis spectra of FA-PEG-COOH, PEI.NH_2-PA, PEI.NH_2-(PEG-FA)-PA and PEI.NH_2-FI-(PEG-FA)-PA dissolved in water, respectively.

As depicted in Figure S2, the UV-vis spectra of FA-PEG-COOH, PEI.NH_2-(PEG-FA)-PA, and PEI.NH_2-FI-(PEG-FA)-PA exhibited distinct absorption peaks at 278 and 360 nm, which correspond to the UV-vis characteristic absorption peaks of FA. The result confirms the successful modification of FA onto the polyethylenimine derivatives. Furthermore, the UV-vis spectrum of PEI.NH_2-FI-(PEG-FA)-PA revealed the presence of characteristic peaks of PA-COOH (265 nm) and FI (511 nm), indicating the successful conjugation of PA-COOH and FI.

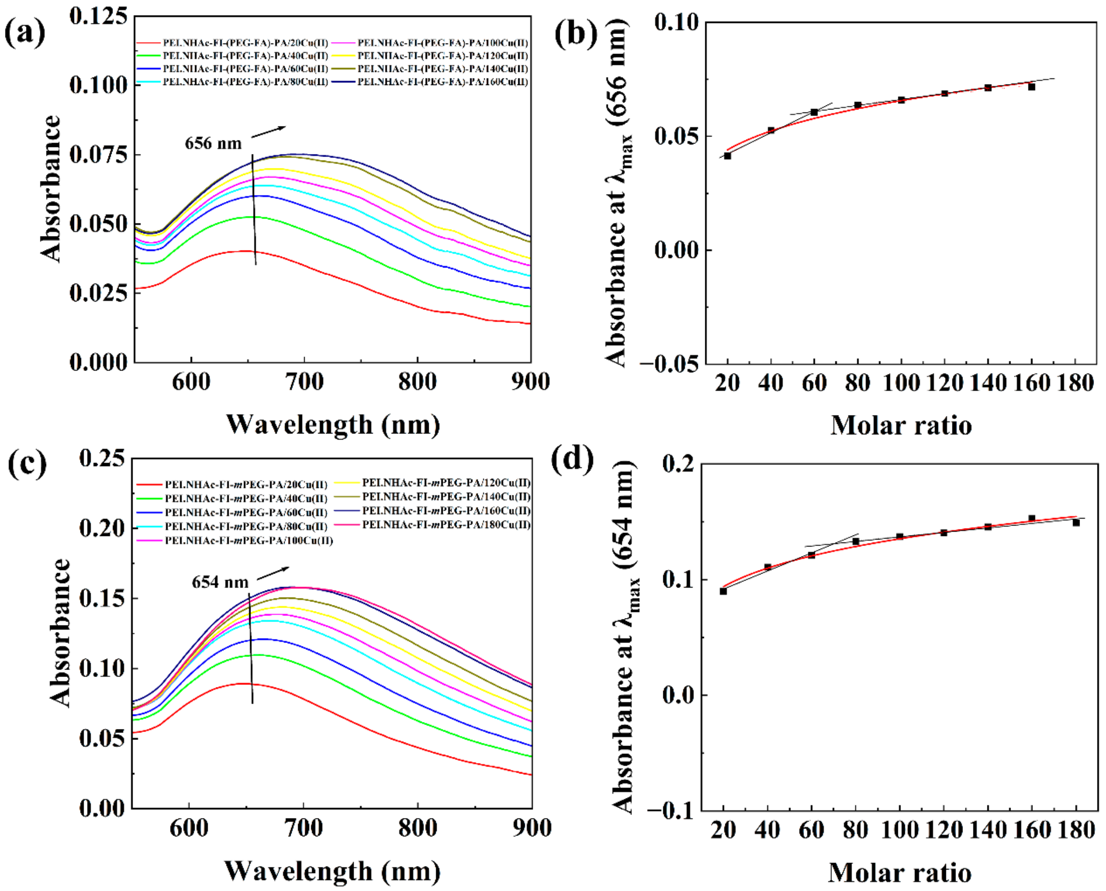

Figure S3. UV-vis spectra of (**a**) PEI.NHAc-FI-(PEG-FA)-PA, (**c**) PEI.NHAc-FI-*m*PEG-PA complexed with different molar equivalents of Cu(II), respectively. The fitted curve of the absorbance at certain wavelength versus the molar ratio (**b**) between Cu(II) and PEI.NHAc-FI-(PEG-FA)-PA, and the molar ratio (**d**) between Cu(II) and PEI.NHAc-FI-*m*PEG-PA.

By analyzing the UV-vis spectra of PEI.NHAc-FI-(PEG-FA)-PA/Cu(II) complexes and PEI.NHAc-FI-*m*PEG-PA/Cu(II) complexes with different molar equivalents of Cu(II) (20-180 molar equivalents of Cu(II) per PEI.NH_2), the Cu(II) complexing capacity of PEI.NHAc-FI-(PEG-FA)-PA and PEI.NHAc-FI-*m*PEG-PA can be determined. After complexing with Cu(II), the absorbance of characteristic peak (656 nm) of PEI.NHAc-FI-(PEG-FA)-PA/Cu(II) complexes increased with the increase of Cu(II) concentration. However, the absorption peak was red-shifted when the molar equivalents of Cu(II) complexed exceeded 60 (Figure S3a). Through fitting analysis, complexing with

more than 60 molar equivalents of Cu(II) per PEI.NH_2 led to the level-off of the absorbance. Therefore, the optimal complexing equivalent of Cu(II) in PEI.NHAc-FI-(PEG-FA)-PA/Cu(II) complexes was quantified to be 60 (Figure S3b). The same change trend of absorption peak was observed in the UV-vis spectra of PEI.NHAc-FI-*m*PEG-PA/Cu(II) complexes, which revealing the increased absorbance of characteristic peak (654 nm) with the increase of Cu(II) concentration. After complexing with more than 60 molar equivalents of Cu(II), the absorption peak of PEI.NHAc-FI-*m*PEG-PA/Cu(II) was red-shifted (Figure S3c). Finally, the optimal complexing equivalent of Cu(II) in PEI.NHAc-FI-*m*PEG-PA/Cu(II) complexes was quantified to be 60-70 *via* fitting analysis (Figure S3d). Based on the comprehensive analysis, 60 molar equivalents of Cu(II) can be determined to be the optimal amount to generate the stable Cu(II)-based nanocomplexes for further study.

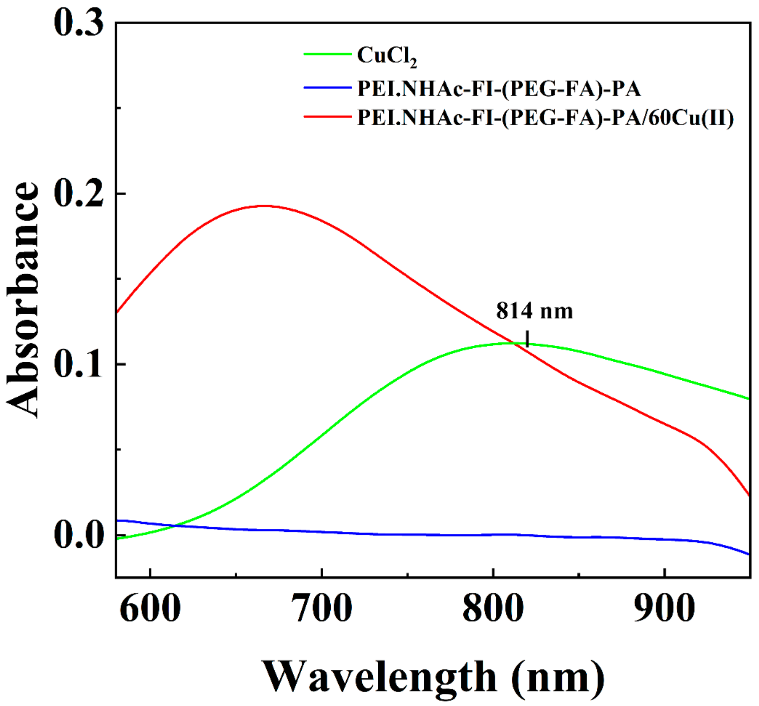

Figure S4. UV-vis spectra of $CuCl_2$, PEI.NHAc-FI-(PEG-FA)-PA, and PEI.NHAc-FI-(PEG-FA)-PA/60Cu(II) dissolved in water.

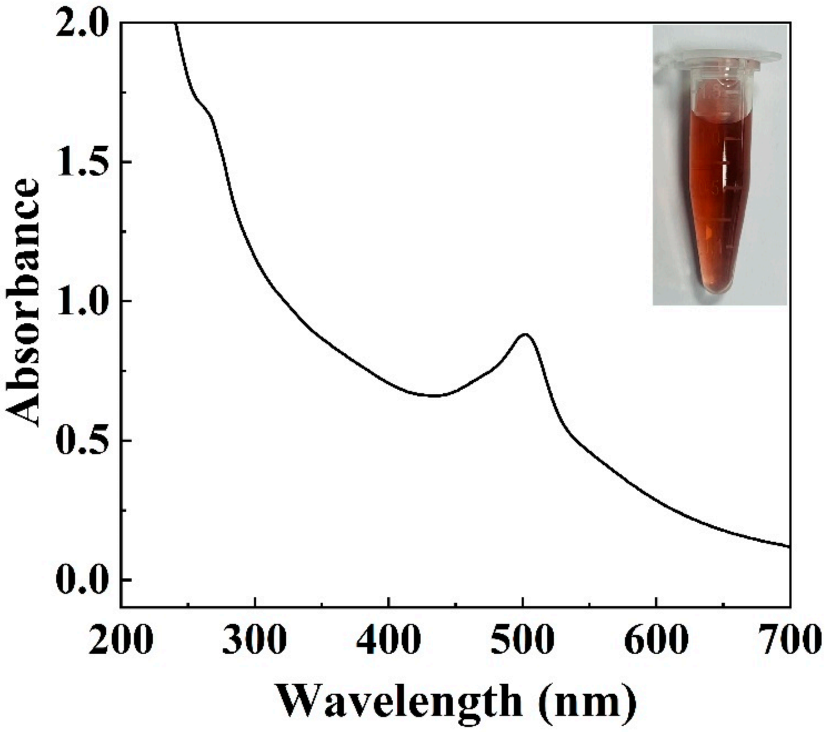

Figure S5. UV-vis spectrum and photograph of FA-Au/Cu(II) PENPs dissolved in water.

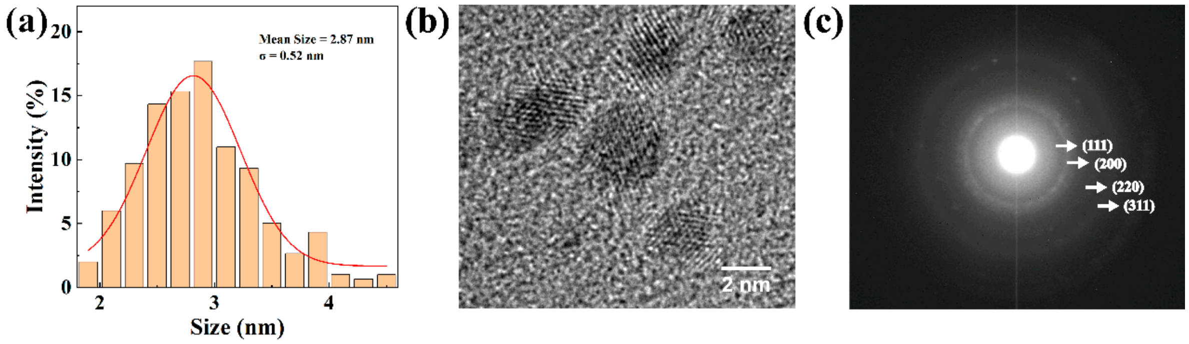

Figure S6. (**a**) Histogram of particle size distribution, (**b**) high resolution TEM image, and (**c**) selected area electron diffraction (SAED) pattern of FA-Au/Cu(II) PENPs.

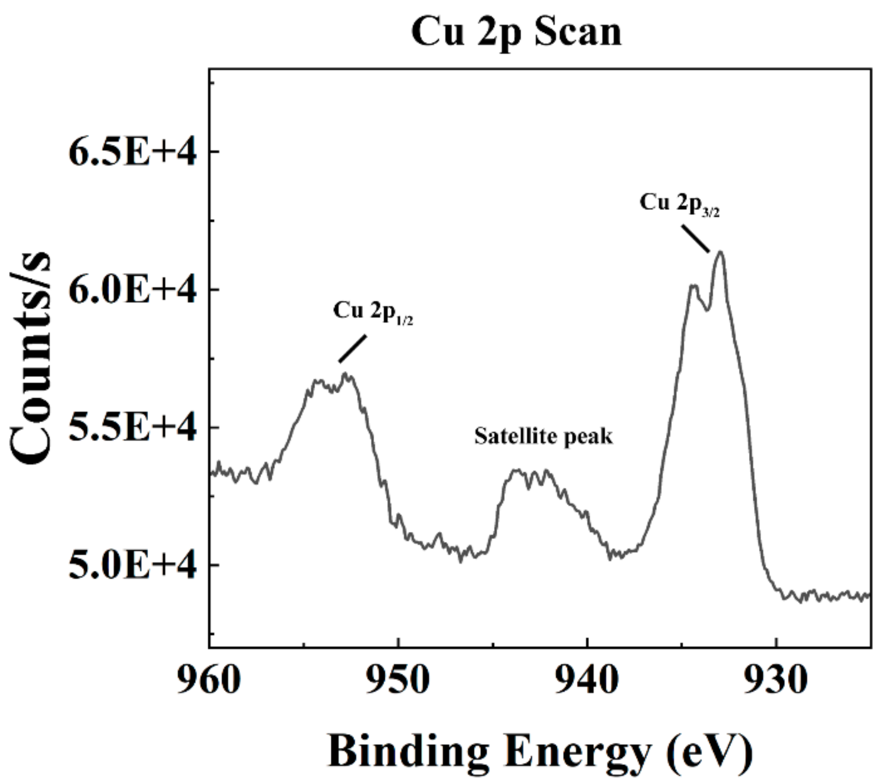

Figure S7. XPS spectrum of Cu 2p region of FA-Au/Cu(II) PENPs.

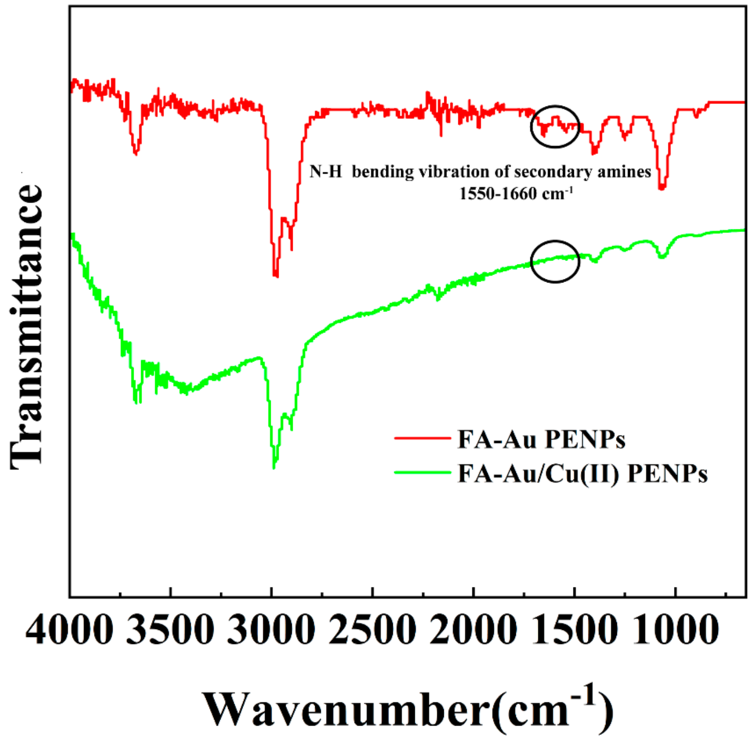

Figure S8. FT-IR spectra of FA-Au PENPs and FA-Au/Cu(II) PENPs.

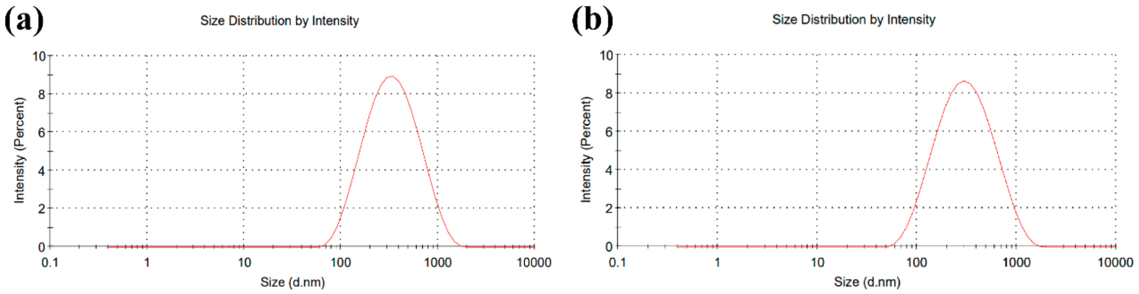

Figure S9. Hydrodynamic size distributions of (**a**) FA-Au PENPs and (**b**) FA-Au/Cu(II) PENPs.

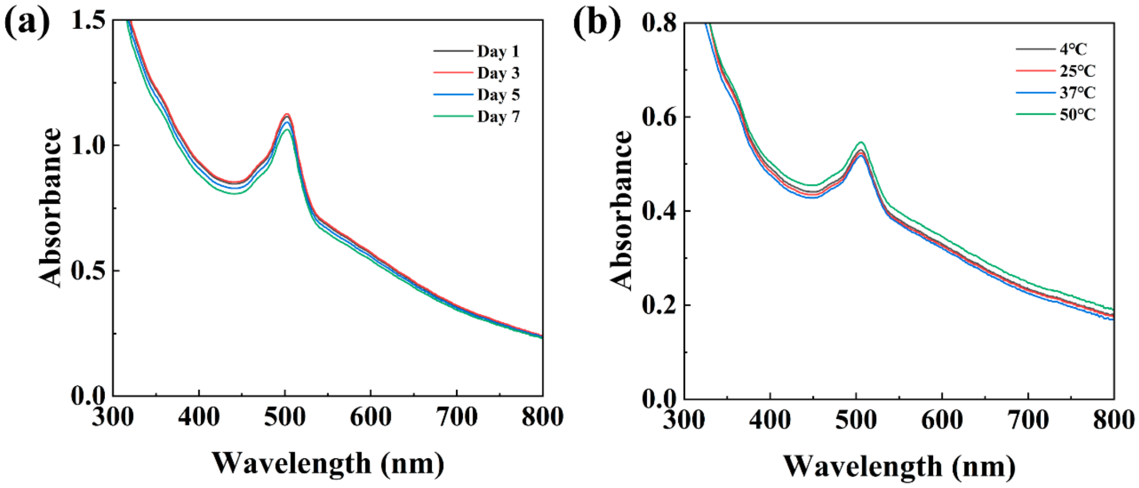

Figure S10. UV-vis spectra of FA-Au/Cu(II) PENPs (**a**) at different time points and (**b**) under different temperature conditions, respectively.

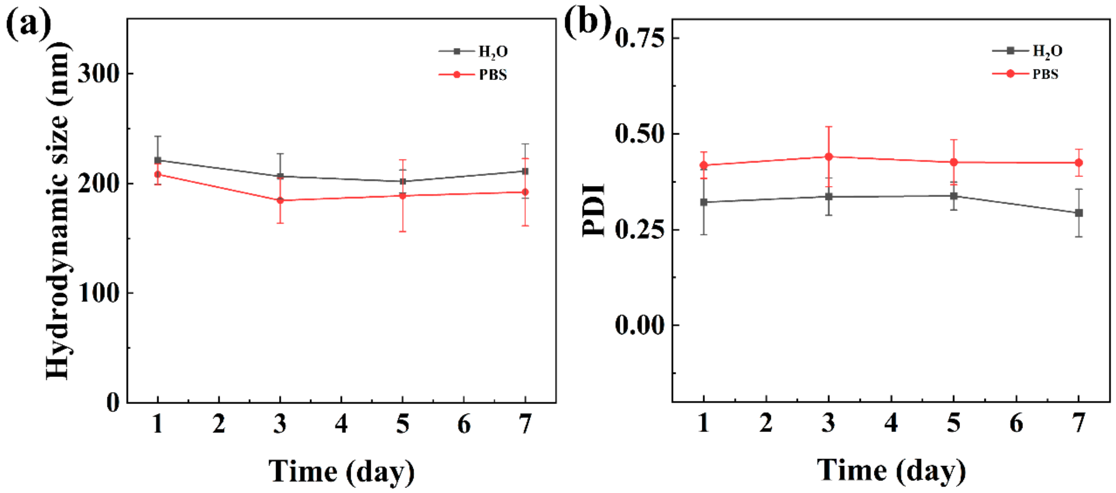

Figure S11. (**a**) Hydrodynamic sizes and (**b**) PDI of FA-Au/Cu(II) PENPs dissolved in H_2O and PBS at different time points, respectively.

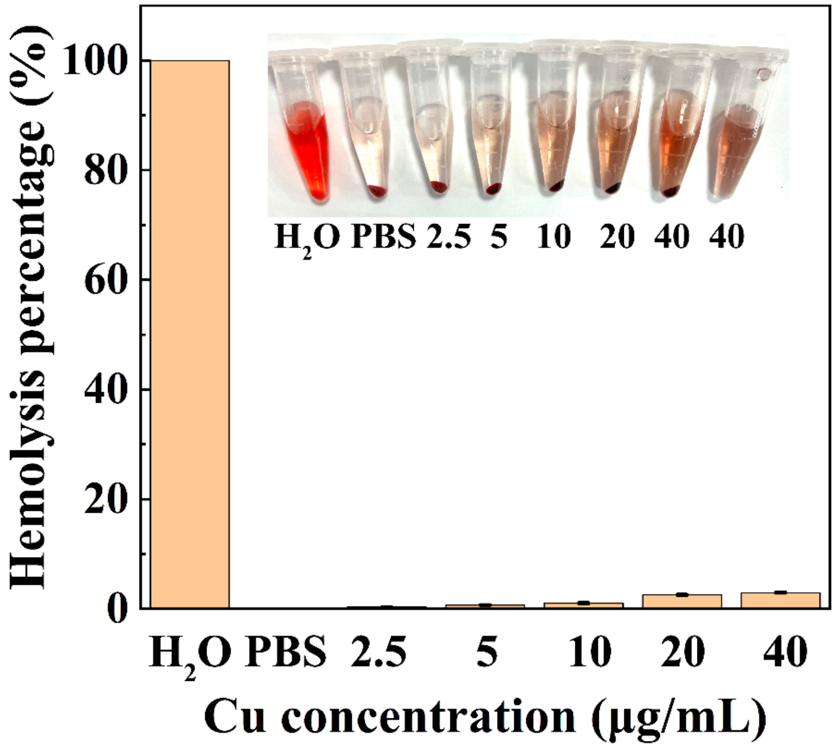

Figure S12. Hemolysis rate and the photograph of red blood cells after being treated with different Cu concentrations of FA-Au/Cu(II) PENPs for 2 h. The red blood cells treated with H_2O and PBS were used as positive and negative controls, respectively. The centrifuge tube containing FA-Au/Cu(II) PENPs ([Cu] = 40 μg/mL) solution without red blood cells is shown on the rightmost side of the photograph.

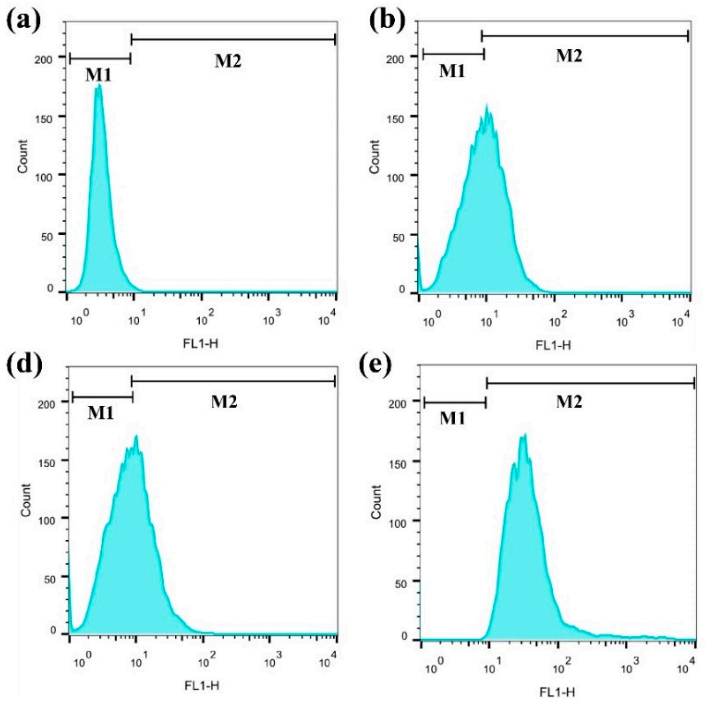

Figure S13. Flow cytometric analysis of (**a**) 4T1-HFAR cells treated with PBS, (**b**) 4T1-LFAR and (**c**) 4T1-HFAR cells treated with Au/Cu(II) PENPs, (**d**) 4T1-LFAR and (**e**) 4T1-HFAR cells treated with FA-Au/Cu(II) PENPs for 2 h, respectively.

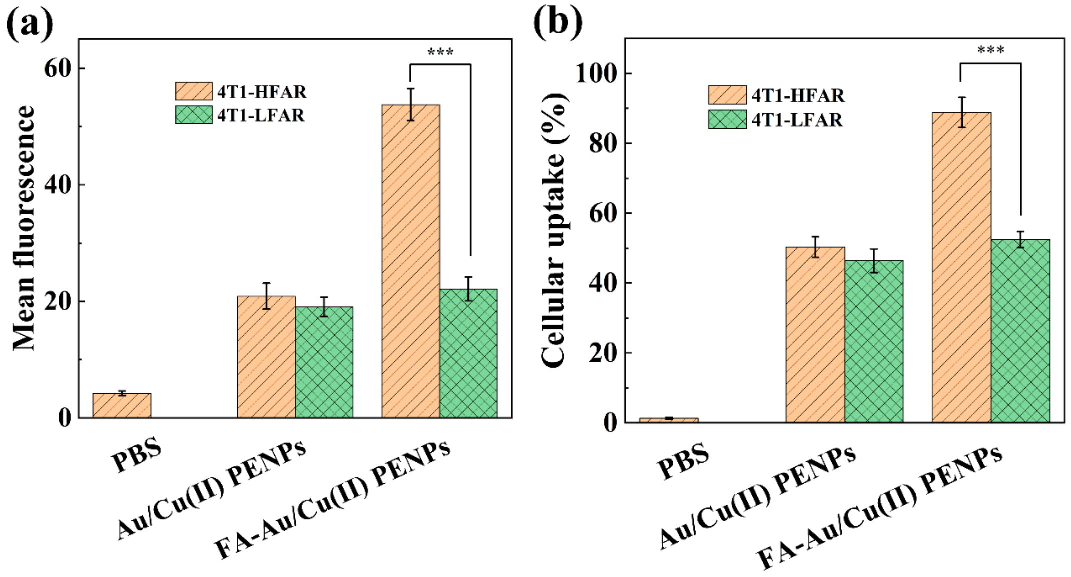

Figure S14. Flow cytometric determination of (**a**) the mean fluorescence and (**b**) the percentage of 4T1 cells uptaken with the nanocomposites after different treatments for 2 h, respectively. (*** $p < 0.001$)

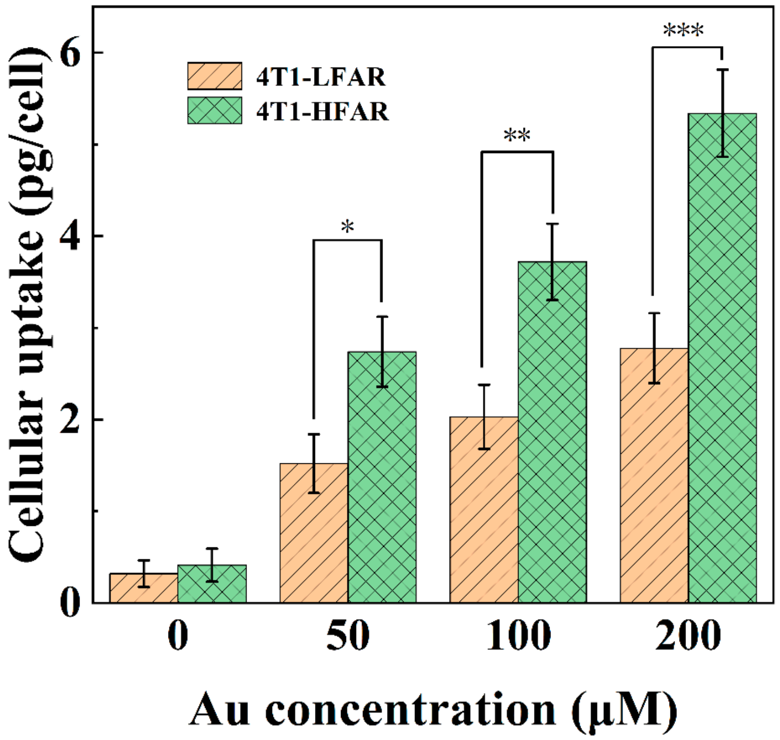

Figure S15. ICP-OES result of Au uptake by 4T1-LFAR and 4T1-HFAR cells treated with FA-Au/Cu(II) PENPs at different Au concentrations (0, 50, 100, 200 μM) for 2 h, respectively. (* $p < 0.05$, ** $p < 0.01$, and *** $p < 0.001$)

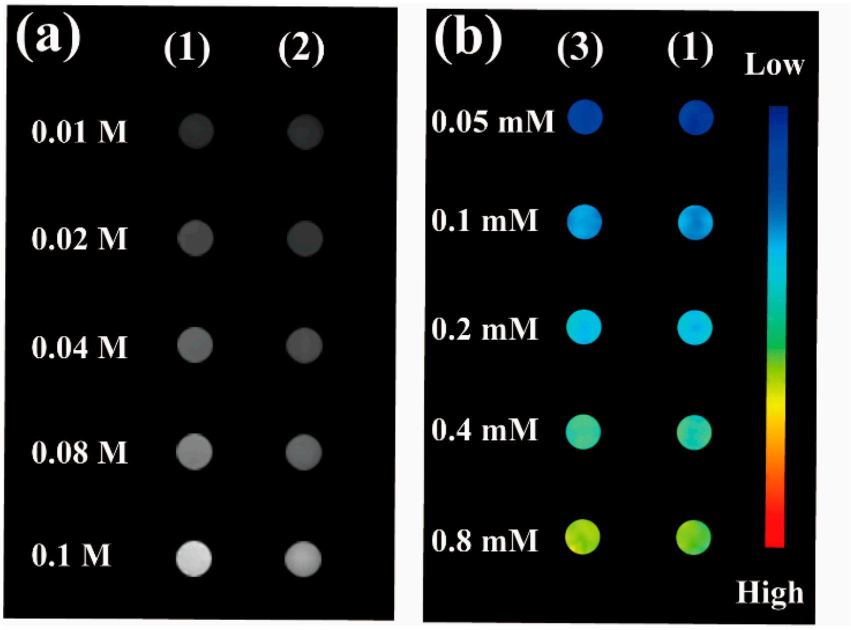

Figure S16. (**a**) CT images of (1) FA-Au/Cu(II) PENPs and (2) Omnipaque at different Au or I concentrations, (**b**) T_1-weighted MR pseudo-color images of (1) FA-Au/Cu(II) PENPs and (3) $CuCl_2$ at different Cu concentrations.

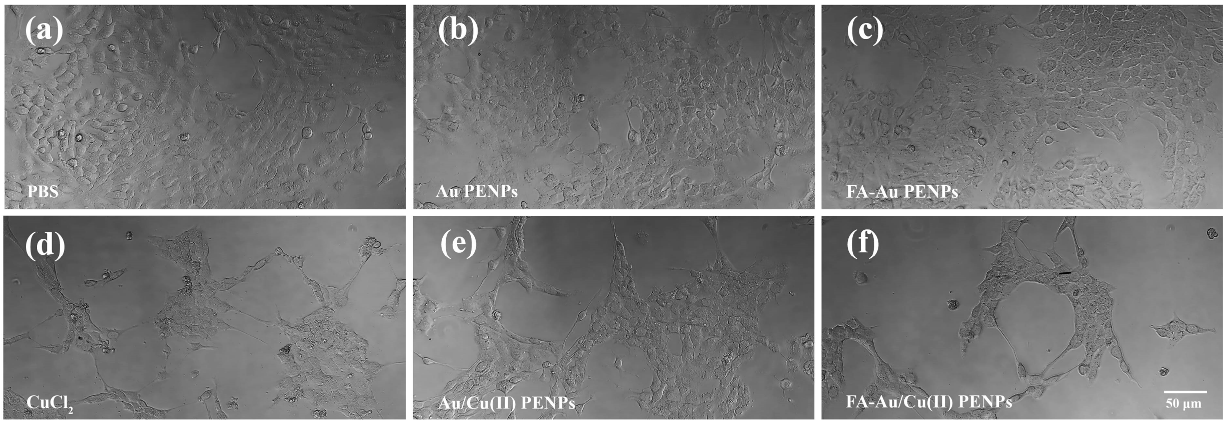

Figure S17. Inverted microscopic images of 4T1 cells after 24 h incubation with (**a**) PBS, (**b**) Au PENPs and (**c**) FA-Au PENPs at PEI.NH_2 concentration of 16 μM, (**d**) $CuCl_2$, (**e**) Au/Cu(II) PENPs and (**f**) FA-Au/Cu(II) PENPs at Cu concentration of 1000 μM, respectively. The scale bar represents 50 μm.

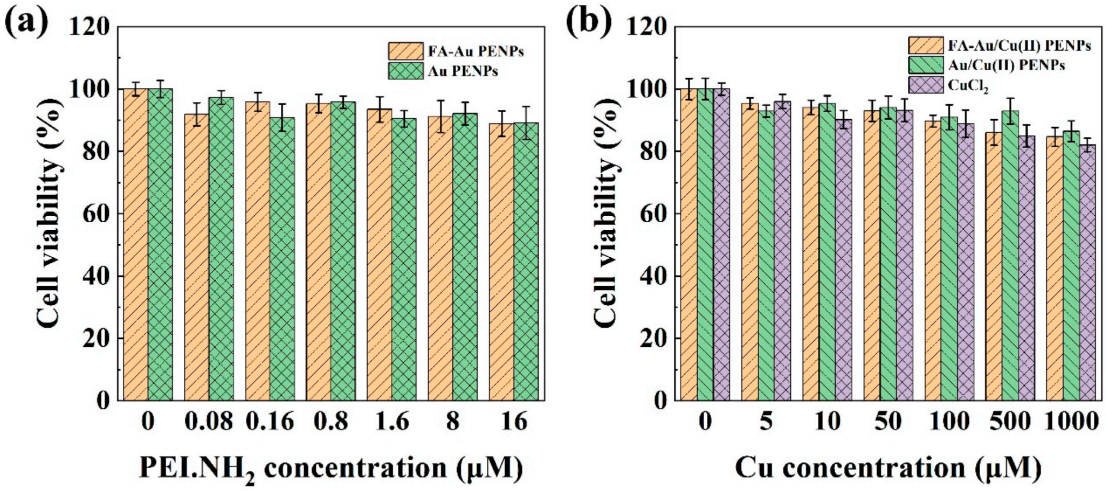

Figure S18. Cell viability of L929 cells after 24 h incubation with (**a**) FA-Au PENPs, Au PENPs at different PEI.NH_2 concentrations and (**b**) FA-Au/Cu(II) PENPs, Au/Cu(II) PENPs, and $CuCl_2$ at different Cu concentrations.

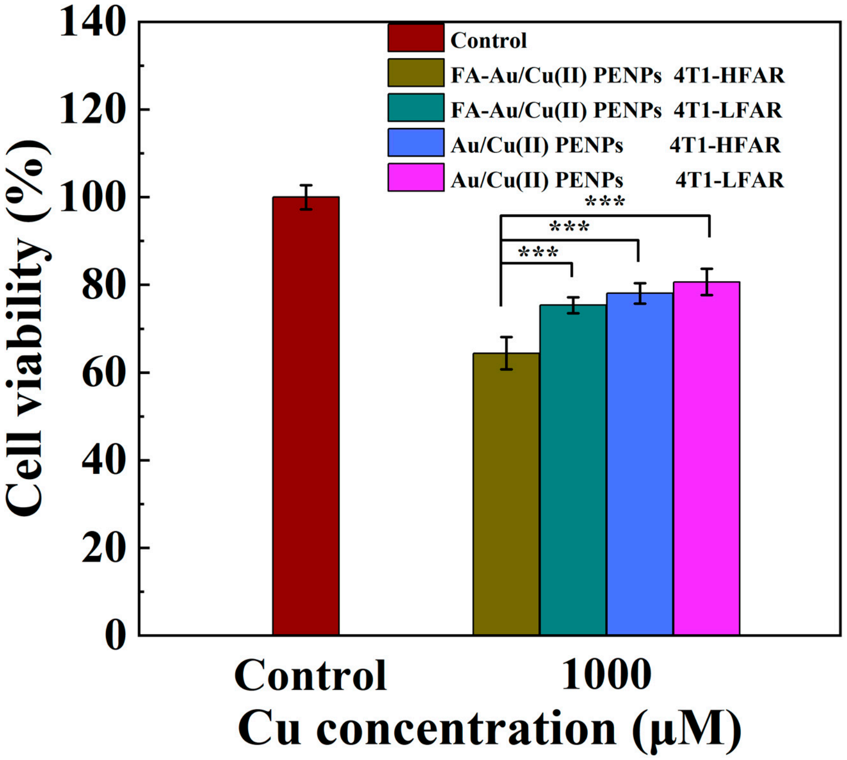

Figure S19. Cell viability of 4T1-LFAR and 4T1-HFAR cells with 2h incubation of FA-Au/Cu(II) PENPs and Au/Cu(II) PENPs at Cu concentration of 1000 μM, followed by 24 h incubation in fresh medium. PBS-incubated 4T1-HFAR cells were set as control. (*** $p < 0.001$)

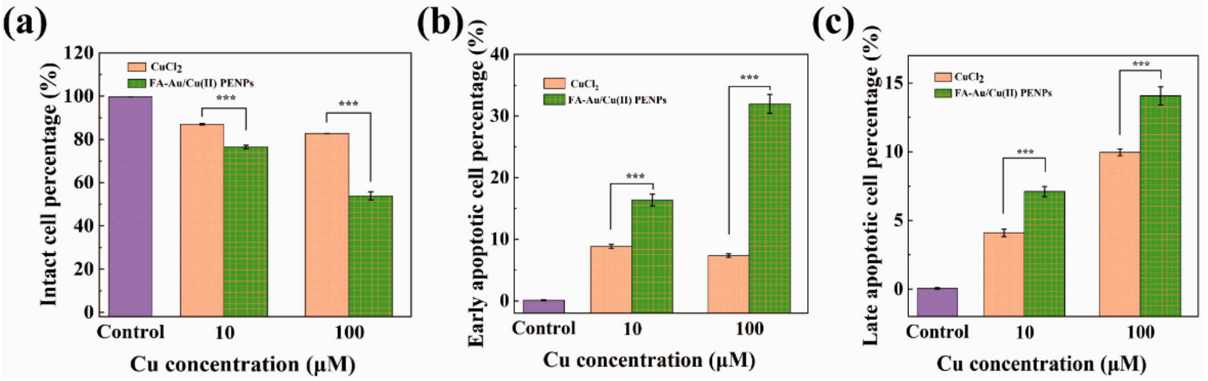

Figure S20. The flow cytometric assay performed using Annexin V-FITC/PI double staining of 4T1 cells after 24 h incubation with different Cu concentrations of $CuCl_2$ and FA-Au/Cu(II) PENPs. The percentages of (**a**) intact cells, (**b**) early apoptotic cells, and (**c**) late apoptotic cells were recorded, respectively. (*** $p < 0.001$)

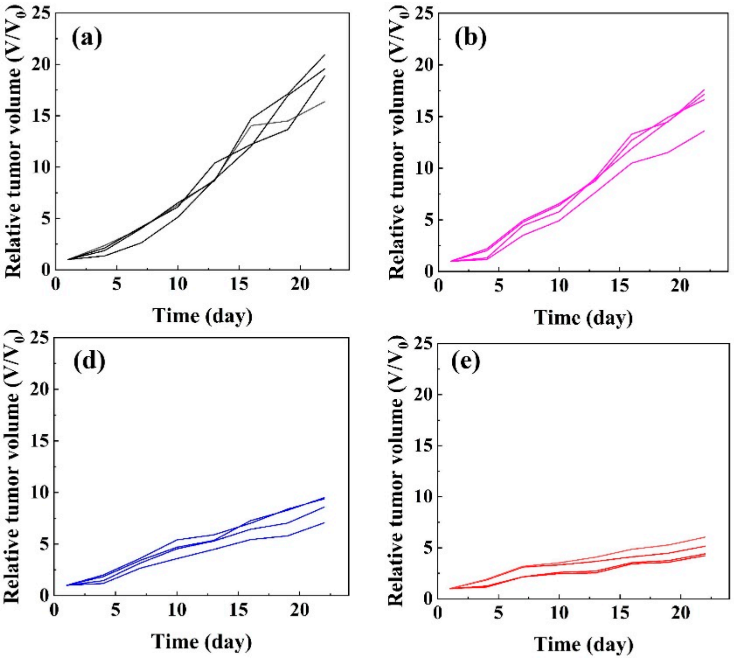

Figure S21. Individual tumor growth curves of 4T1-tumor bearing mice treated with (**a**) NS, (**b**) Au PENPs, (**c**) FA-Au PENPs, (**d**) Au/Cu(II) PENPs, and (**e**) FA-Au/Cu(II) PENPs, respectively.

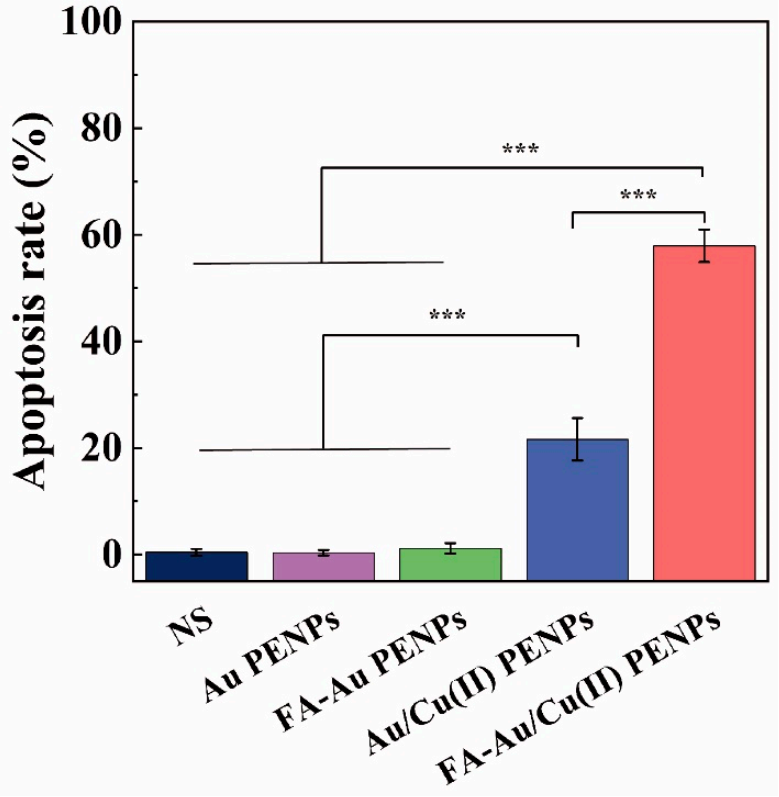

Figure S22. The apoptosis rate of tumor tissues with different treatments recorded from TUNEL stained sections. (*** $p < 0.001$)

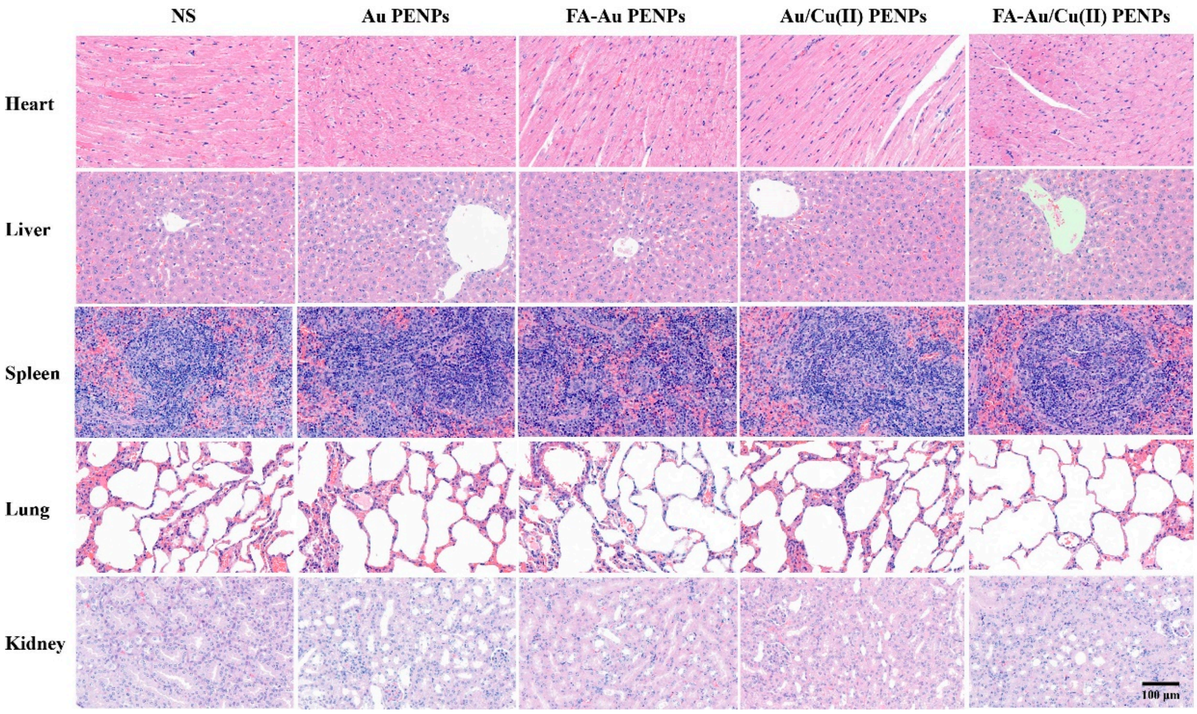

Figure S23. H&E staining of the major organs of 4T1 tumor-bearing mice after treatment with different nanoparticles, respectively. The scale bar represents 100 μm.

References

1. Zhu, J.Y.; Zheng, L.F.; Wen, S.H.; Tang, Y.Q.; Shen, M.W.; Zhang, G.X.; Shi, X.Y. Targeted cancer theranostics using alpha-tocopheryl succinate-conjugated multifunctional dendrimer-entrapped gold nanoparticles. *Biomaterials* **2014**, *35*, 7635-7646.

2. Zhu, J.; Zhao, L.; Zhao, P.; Yang, J.; Shi, J.; Zhao, J. Charge-conversional polyethylenimine-entrapped gold nanoparticles with ^{131}I-labeling for enhanced dual mode SPECT/CT imaging and radiotherapy of tumors. *Biomater. Sci.* **2020**, *8*, 3956-3965.

3. Song, C.; Ouyang, Z.; Gao, Y.; Guo, H.; Wang, S.; Wang, D.; Xia, J.; Shen, M.; Shi, X. Modular design of multifunctional core-shell tecto dendrimers complexed with copper(II) for MR imaging-guided chemodynamic therapy of orthotopic glioma. *Nano Today* **2021**, *41*, 101325.

4. Fan, Y.; Zhang, J.; Shi, M.; Li, D.; Lu, C.; Cao, X.; Peng, C.; Mignani, S.; Majoral, J.-P.; Shi, X.

Poly(amidoamine) dendrimer-coordinated copper(II) complexes as a theranostic nanoplatform for the radiotherapy-enhanced magnetic resonance imaging and chemotherapy of tumors and tumor metastasis. *Nano Lett.* **2019**, *19*, 1216-1226.

5. Zhu, J.Y.; Wang, G.; Alves, C.S.; Tomas, H.; Long, Z.J.; Shen, M.W.; Rodrigues, J.; Shi, X.Y. Multifunctional dendrimer-entrapped gold nanoparticles conjugated with doxorubicin for pH-responsive drug delivery and targeted computed tomography imaging. *Langmuir* **2018**, *34*, 12428-12435.
6. Hao, Y.C.; Gao, Y.; Fan, Y.; Zhang, C.C.; Zhan, M.S.; Cao, X.Y.; Shi, X.Y.; Guo, R. A tumor microenvironment-responsive poly(amidoamine) dendrimer nanoplatform for hypoxia-responsive chemo/chemodynamic therapy. *J. Nanobiotechnol.* **2022**, *20*, 43.
7. Wang, L.; Xu, Y.T.; Liu, C.; Si, W.L.; Wang, W.J.; Zhang, Y.W.; Zhong, L.P.; Dong, X.C.; Zhao, Y.X. Copper-doped MOF-based nanocomposite for GSH depleted chemo/photothermal/chemodynamic combination therapy. *Chem. Eng. J.* **2022**, *438*, 135567.
8. Cai, X.Y.; Cai, D.; Wang, X.Z.; Zhang, D.; Qiu, L.; Diao, Z.Y.; Liu, Y.; Sun, J.B.; Cui, D.X.; Liu, Y.L.; et al. Manganese self-boosting hollow nanoenzymes with glutathione depletion for synergistic cancer chemo-chemodynamic therapy. *Biomater. Sci.* **2024**, *12*, 3622-3632.
9. Shi, X.; Wang, S.; Meshinchi, S.; Van Antwerp, M.E.; Bi, X.; Lee, I.; Baker, J.R., Jr. Dendrimer-entrapped gold nanoparticles as a platform for cancer-cell targeting and imaging. *Small* **2007**, *3*, 1245-1252.
10. Fan, Y.; Tu, W.Z.; Shen, M.W.; Chen, X.M.; Ning, Y.S.; Li, J.J.; Chen, T.F.; Wang, H.; Yin, F.F.; Liu, Y.; et al. Targeted tumor hypoxia dual-mode CT/MR imaging and enhanced radiation therapy using dendrimer-based nanosensitizers. *Adv. Funct. Mater.* **2020**, *30*, 1909285.
